# Revisiting the missing protein-coding gene catalog of the domestic dog

**DOI:** 10.1186/1471-2164-10-62

**Published:** 2009-02-04

**Authors:** Thomas Derrien, Julien Thézé, Amaury Vaysse, Catherine André, Elaine A Ostrander, Francis Galibert, Christophe Hitte

**Affiliations:** 1 Institut de Génétique et Développement, CNRS UMR6061, Université de Rennes1, 2 Av du Pr. Léon Bernard, 35043 Rennes, France; 2 Cancer Genetics Branch, National Human Genome Research Institute, National Institutes of Health, 50 South Drive, Bethesda MD 20892, USA; 3 Centre for Genomic Regulation (CRG), Bioinformatics Program C/Dr. Aiguader, 88 08003 Barcelona, Spain

## Abstract

**Background:**

Among mammals for which there is a high sequence coverage, the whole genome assembly of the dog is unique in that it predicts a low number of protein-coding genes, ~19,000, compared to the over 20,000 reported for other mammalian species. Of particular interest are the more than 400 of genes annotated in primates and rodent genomes, but missing in dog.

**Results:**

Using over 14,000 orthologous genes between human, chimpanzee, mouse rat and dog, we built multiple pairwise synteny maps to infer short orthologous intervals that were targeted for characterizing the canine missing genes. Based on gene prediction and a functionality test using the ratio of replacement to silent nucleotide substitution rates (*d*_N_/*d*_S_), we provide compelling structural and functional evidence for the identification of 232 new protein-coding genes in the canine genome and 69 gene losses, characterized as undetected gene or pseudogenes. Gene loss phyletic pattern analysis using ten species from chicken to human allowed us to characterize 28 canine-specific gene losses that have functional orthologs continuously from chicken or marsupials through human, and 10 genes that arose specifically in the evolutionary lineage leading to rodent and primates.

**Conclusion:**

This study demonstrates the central role of comparative genomics for refining gene catalogs and exploring the evolutionary history of gene repertoires, particularly as applied for the characterization of species-specific gene gains and losses.

## Background

Comparative genomics plays a key role in understanding organism evolution, refining functional annotation and identifying orthology relationships. By taking advantage of whole-genome sequence assemblies with a high level of coverage [1-4], one can seek to provide exhaustive and genome-scale level predictions regarding functional sequence [5]. The general approach relies on the exploitation of sequence similarities [6-8] phylogenetic data [9,10], evolutionary models [11,12] and evidence regarding conservation of gene order [13-15]. These often complementary comparative approaches have been developed to estimate and improve the identification of functional sequences for both newly sequenced species as well as reference species, such as human and mouse [16-18]. Moreover, multispecies genome scale comparisons allow to refine protein-coding genes annotation [19-21] as well as better understanding of the timing and the frequency of duplication events for lineage-specific genes called in-paralogs [22,23].

Fine-scale comparative maps constructed using robust orthologous sequences are key for allowing identification, visualization and characterization of conserved segments as well as collinearity of gene order between the species [24,25]. Gene order between species is not random and this has been shown to correlate with, for example, co-expressed and co-regulated genes suggesting a functional significance [26]. Otherwise, gene order conservation between species could also be exploited to identify relocated protein-coding genes in non-syntenic chromosomal regions [27], as well as potentially retrotransposed genes given that the latter correspond mostly to pseudogenes inserted in non-syntenic regions [10]. Consequently, as part of the characterization of architecture of a genome, analysis of gene order conservation between species can be a strong indicator for both gene prediction [28] and identification of gene loss [29].

In this study, we have analyzed the sequence assembly of the domestic dog for which the annotation process identified less protein-coding genes than expected compared to predictions from the primates and rodent genomes. We focused on a set of 412 genes that are all annotated in four closely related mammals; human, chimpanzee, mouse and rat, but absent in the dog genome in the most recent assembly of the dog (CanFam 2.0). We exploited the property of gene adjacency conservation between related species to target in-depth sequence alignments over a short genomic interval. In addition, our approach includes a functionality test that investigates the ratio of amino acid replacement (nonsynonymous, *d*_N_) to silent (synonymous, *d*_S_) substitution rates, which indicates selective constraints acting on a given genomic regions [10]. As mutations in genes causing amino acid replacements with functional consequences are selected against in contrast to mutations occurring in pseudogenes, we took advantage of the distinctive patterns of *d*_N_/*d*_S _ratios to refine the identification of new gene predictions and gene losses occurring in dog.

Using the above strategies we identified 232 canine genes for which synteny conservation, cross-species sequence analysis and the neutral rate of evolution based on *d*_N_/*d*_S _results converged strongly to support their existence. In addition, we identified 69 gene-loss candidates of which predictions for which accumulating ORF-disrupting mutations, and significant *d*_N_/*d*_S _ratios support scenarios of 21 genes lost as pseudogenes in the canine species. To further characterize gene losses, we inferred their phyletic pattern in ten species from chicken to human over a period of 310 million years. Therefore, we were able to differentiate canine-specific losses from gene losses that have occurred in others lineage or genes formed after the evolutionary branchpoint leading to dog.

## Results

Using all annotated genes from human, chimpanzee, mouse, rat and dog (Ensembl v42) [30], we extracted 412 genes annotated as protein-coding in all species but dog. These genes exhibit a '1:1:1:1:0' phyletic pattern, that is indicative of the presence/absence of genes with a one-to-one orthologous relationship among the five species. We refer to these as 'missing genes' for purposes of this study. We examined the structural features of the 412 missing genes in the four mammalian reference sequences and compared them to an independent and randomly selected set of 400 genes. The mean length of the protein products of the missing genes set was 722 amino acids (AA), which is significantly smaller than the random set at 905 AA (*t *test; *P *= 6.8e - 11). Similarly, the mean transcript size was ~50% smaller than observed in a random set (*t *test; *P *= 2.6e - 9). The mean number of exons in missing genes was also smaller (5.8 vs 9.8; *t *test;*P *= 3.7e - 13) than the random set and particularly single-exon genes were found to be over represented by 15%. To ensure that single-exon missing genes were functional and not processed pseudogenes, we analyzed each, using the human dataset, for accumulated degenerative mutations (frameshifts and premature stop codons) in their coding sequence and found none. In addition, we identified sequence alignment between single-exon genes and ESTs (sequence similarity > 96% for at least 150 bp) for 95% of them.

To test the underlying assumption that missing genes may be implicated in particular biological pathways, we examined their functional annotation in the context of Gene Ontology (GO) using the program GO Tree Machine [31]. Using the human sequence as a reference, the results demonstrate that the missing gene set is enriched for genes implicated in physiological pathways of immunity and organism responses to pathogens (12 genes), olfaction (16) and regulation of transcription (63). This classification comprises functional pathways that play an important role in the adaptation of organisms to their environment. Interestingly, these biological functions are often linked to large proteins families that are attractive targets for lineage-specific functions and lineage-specific loss and gain of genes [32].

### Constructing synteny maps with 1:1 orthologs

We extracted pairwise sets of 14,997; 14,798; 14,667 and 14,065 one-to-one (1:1) orthologous protein-coding genes (Ensembl v42) between human and dog (H-D), chimpanzee-dog (C-D), mouse-dog (M-D) and rat-dog (R-D), respectively. Using those 1:1 orthologs as comparative anchors, we built four fine-scale whole-genome pairwise synteny maps (Additional data file 1) with the program AutoGRAPH, which we recently developed [13]. We identified 218, 229, 326 and 325 CSOs, i.e. chromosomal segments for which markers are in the same linear order on the chromosome as noted across species [25], between H-D, C-D, M-D and R-D respectively. The mean distance between two consecutive genes was ~180 kb. In all synteny maps, CSOs cover almost the entire genome while breakpoint regions, areas delimitating CSOs, cover only ~5% of a genome and may contain single-gene segment or very short synteny blocks [33] (Additional data file 2).

**Figure 1:**
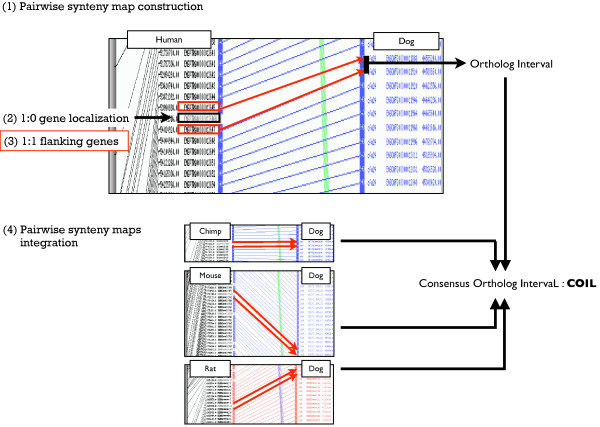
**Consensus Ortholog IntervaL identification**. The figure illustrates the 4-step method to infer targeted interval for gene prediction. (1) is the first step that build the pairwise synteny map (here a schematic Human-dog syntenic map) using 1:1 orthologs that are connected through colored lines. (2) 1:0 gene ('missing gene' in the dog) is positioned on the reference species of the synteny map. (3) indicates the identification of flanking 1:1 orthologs used to define an orthologous interval on the canine chromosome as indicated by red arrows. (4) is the last step that integrates the four orthologous intervals using all pairwise synteny maps (Chimpanzee-dog; Mouse-dog and Rat-dog) to define a Consensus Ortholog IntervaL (COIL) as shown on the right of the figure.

In each pairwise synteny map, we localized the missing gene orthologs on the reference sequence (Figure 1). Of the 412 missing genes, the vast majority (mean of 92.3%; range 92 to 94%) mapped within CSOs with only 7.7% mapping within breakpoints. In all reference species the missing genes spanned all chromosomes, although their distribution varied greatly, i.e. one to 44 per human (HSA) chromosome in the case of the human-dog synteny map.

### Targeting genomic intervals

We used multiple pairwise synteny maps described above to identify short, targeted, orthologous genomic intervals. On each reference genome, these intervals are delimited by the closest flanking 1:1 orthologs on either side of each missing gene that in turn define orthologous intervals on the canine genome as shown in Figure 1. The use of multiple pairwise maps enabled us to identify the shortest consensus interval on the canine genome to search for genes, that we refer to as Consensus Ortholog IntervaLs (COILs) (Figure 1). From the 412 missing genes, we delimited 383 COILs (92.9%) having a mean size of 347 kb (Additional data file 3). For a set of 17 COILs (4.1%) localized in common breakpoint regions (i.e. overlapping between at least two species) [24,34] and for 12 missing genes, no COIL could be determined because of the absence of a consensus interval.

### Targeted gene prediction

**Figure 2:**
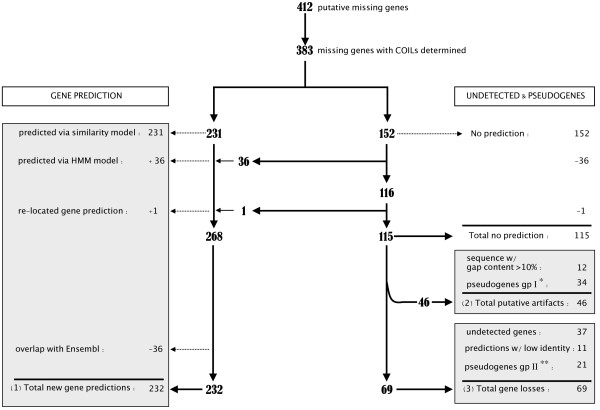
**Flowchart of the computational analysis**. The left pipeline indicates all steps in the computational analysis of gene predictions and the right pipeline shows a detailed account of the process of undetected genes and pseudogenes. Gray boxes summarize the three main categories (1) new gene predictions, (2) putative artifacts, * indicates pseudogenes identified with low confidence (group I), and (3) gene losses, (**) indicates pseudogenes identified with accumulated mutations (group II) and higher *dN/dS *support. See text for details.

Within each canine COIL, we used the GeneWise program [6] to splice and align the protein sequence of each reference species in order to most accurately predict the structure of the dog gene. We retained gene predictions produced by at least two reference species protein templates. This produced 231 gene structure predictions with amino acid identity > 40% (Figure 2). Fifty-three genes were predicted using only rodent protein sequence as templates, thus illustrating the complementary contribution of multispecies analysis. We post-processed GeneWise results to detect potential gene features and found the presence of a coding start site for 53.1% of the gene predictions. In addition, amongst the 231 predicted genes, 75% of the predictions with multi-exonic structure exhibit at least a canonical splice site (GT/AG).

To address the question whether COIL delimitation is too restrictive for gene prediction, we aligned the human transcript sequences corresponding to the 383 missing genes for which we defined a COIL, against the assembly of the canine genome sequence (CanFam 2.0) with the Exonerate program [35]. We repeated the analysis with chimpanzee, mouse and rat transcript sequences. We considered the best five matching sequences to relax the limitations of conventional best-match methods [29]. Then, we defined a concordance between the COIL approach and the whole-genome sequence analysis, when matching sequences from the Exonerate-based analysis for at least two species were totally embedded in COILs. Based on this criterion, concordance was obtained for 342 (89.2%) genes. Of the 41 instances with no agreement between the expected syntenic location and the whole-genome sequence analysis, 36 showed weak match (identity < 20%) within the canine genome assembly suggesting unspecific alignment while five showed a significant match, from at least two species suggesting that these genes may have acquired a new location in the dog. Of the latter five instances, we identified only one gene prediction (*PLA2G4C*) with conservative criteria indicating a relocated gene in a non-syntenic genomic area.

In this study, we applied Genewise program with a sequence similarity-based method that explicitly models the conservation of gene structure and a high degree of conservation. As such model is known to show a marked decrease in performance for less similar genes [36], we further investigate the undetected subset of genes using a probabilistic pair hidden Markov model (HMM) that show a weaker dependence on percent identity and performs better to pick out distant homologs. The Genewise HMM based analysis allowed to predict 36 additional genes (Figure 2). Both prediction sets were merged into a single set (n = 268) for further analysis.

Sequence alignments were next generated between gene predictions and canine transcript sequences (Unigene april 08 [37]). We identified significant alignment (sequence similarity > 96% for at least 150 bp) in 53% of cases with an average of 7.5 ESTs/mRNA per gene prediction (range 1–99). Using Interproscan, [38] protein motifs were found from InterPro database for 80.5% of the gene predictions, providing additional evidence for dog gene identification.

As a further validation step, the construction of canine predicted protein three-dimensional models was investigated based on the homologous structure of the human ortholog or paralog (>40% identity), which was used as a template. For the subset of genes for which the 3D structure is solved (n = 21), canine-human comparative modelling was determined using the SWISS-MODEL server [39]. In 16 instances of canine-human comparative modelling, the mean identity obtained between sequences was 70%. Homology-based 3D model for each canine prediction was validated using the Verify 3D graphs [40] (data not shown) that distinguish between homology models of higher and lower accuracy.

To test for possible overlap between gene predictions obtained in this study and all canine genes annotated in Ensembl (v42), we performed sequence alignment between these two sets of predictions. A total of 232 (88%) predicted genes did not overlap any Ensembl annotated protein-coding genes. Therefore, these were classified as "definite" gene identifications together with the delineation of new orthologous relationships with the four reference species (Additional data file 4). The remaining 36 gene predictions overlapped an annotated gene (protein identity > 80%) indicating that these gene predictions correspond to sequences already defined as genes, but with undetected or spurious orthologous relationships (Figure 2). [41].

### Gene prediction assessment from *dN*/*dS *analysis

**Figure 3:**
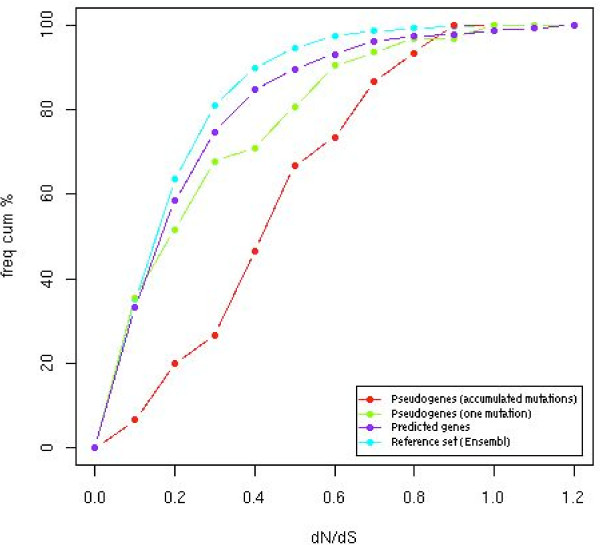
***D*_N_/*d*_S _cumulative frequency distribution of references, gene predictions and pseudogene predictions sets**. Benchmark, predicted genes, pseudogenes (with one mutation) and pseudogenes (with accumulated mutations) sets exhibit a median *d*_N_/*d*_S _of 0.15, 0.18, 0.22, 0.47, respectively, compared to their human functional orthologues. While the *d*_N_/*d*_S _distribution of pseudogenes with accumulated mutations sets is clearly shifted upwards to the theoretical value of 0.57 (average between 1.0 for no selection and 0.15 for selection from the benchmark set), the pseudogene set with one mutation is not significantly shifted suggesting this set may contains spurious pseudogene prediction. Predicted and benchmark gene sets have a similar *d*_N_/*d*_S _cumulative frequency distribution indicating comparable selective constraints level.

**Figure 4:**
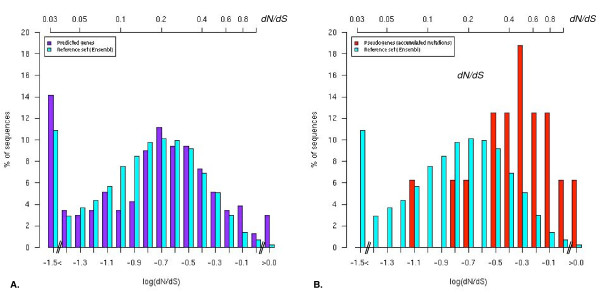
***D*_N_/*d*_S _distributions of benchmark and test sets**. A. The *d*_N_/*d*_S _distribution (as log *d*_N_/*d*_S_) of the test set (new predicted genes) is represented in purple and benchmark set (human-dog 1:1 orthologous) is represented in blue. Test set exhibits a *d*_N_/*d*_S _distribution similar to the benchmark set (Mann-Whitney; *P *= 0.16) suggesting comparable selective constraints for both sets. B. In contrast the *d*_N_/*d*_S _distribution of the pseudogene (with accumulated mutations) set (red) is significantly shifted upwards (Mann-Whitney; *P *= 5.17e - 6) in comparison to the benchmark set, indicating relaxation of selective constraints on the predicted pseudogenes.

To assess the validity of gene predictions through the strength and direction of selective constraints, we used a functionality test that uses the ratio of replacement to silent nucleotide substitution rates (*d*_N_/*d*_S_). The ratio *d*_N_/*d*_S_, where *d*_N _is the number of non-synonymous nucleotide substitution per non-synonymous site and *d*_S _the number of synonymous nucleotide substitution per synonymous site, is used as a proxy for the evolutionary constraints that occur on nucleotide substitution [42]. The calculation of the *d*_N_/*d*_S _ratio requires the comparison to a homologous reference sequence. First, we constructed a benchmark set of true orthologous genes using all 1:1 orthologous genes between human and dog (n = 14,994) to obtain a representative *d*_N_/*d*_S _value. From this benchmark set, we calculated the median *d*_N_/*d*_S _ratio of 0.15 using all *d*_N_/*d*_S _values extracted from the pairwise alignments of transcripts (Figure 3). To assess the 232 gene predictions identified in this study with the functionality test, we determined *d*_N_/*d*_S _ratio for each of the gene predictions in comparison to their human functional orthologous gene from pairwise transcripts alignments. We calculated a median *d*_N_/*d*_S _of 0.19, a value highly similar to the benchmark set (0.15). To further assess the *d*_N_/*d*_S_comparison, *d*_N_/*d*_S _values were analyzed through their distributions (as log *d*_N_/*d*_S_) between benchmark and predicted genes sets (Figure 4) and we did not detect statistically significant differences (Mann-Whitney test; *P *= 0.16). Therefore *d*_N_/*d*_S _similar distributions are indicative of similar high selective constraints and little or no positive selection on both benchmark and predicted genes sets, suggesting the functional properties of the canine gene predictions products involved are conserved.

To analyze the evolutionary rate of the new canine predicted gene sequences in a phylogenetic context we used the 232 mouse genes in addition to human genes and dog predicted genes to assess the levels of selective constraint of each lineage in comparison to the rest of the tree. In this way, differences or similarity in selective constraints can be predicted on all lineages within the phylogeny. For each of the 232 genes, we inferred the *d*_N _and *d*_S _values and calculated the *d*_N_/*d*_S _ratio. The median *d*_N_/*d*_S _for the dog lineage was found between human and mouse (Table 1), a result in agreement to these determined for 13,816 human, mouse and dog genes with 1:1:1 orthologs [2] with similar differences found across the three lineages.

**Table 1:** Median and mean *dS *and *dN/d*S values of pseudogenes, predicted genes and reference set of human-canine orthologues

value	Pseudogenes with one mutation	Pseudogenes with several mutations	Predicted genes	Benchmark set 1:1 dog-human orthologs
*dS *median	0.45	0.44	0.39	0.39

*dS *mean	0.48	0.46	0.40	0.38

*dN/dS *median	0.18	0.50	0.19	0.15

*dN/dS *mean	0.28	0.50	0.26	0.20

### Pseudogene predictions

Off the 412 missing genes, a subset of 55 predictions containing ORF-disrupting mutations lead to pseudogene identification. Among pseudogenes, we determined if protein sequences have different numbers of in-frame stop codons and/or frameshift disruptions. Using such quantitative measures, two mutation levels were apparent. A set of inactivated genes (n = 21) was predicted with accumulated mutations (mean = 4.2; range 2–11) and a second set (n = 34) was predicted with one mutation (Figure 3). To normalize the mutation rate by taking into account the coding sequence length, we expect proteins of similar lengths to now have similar numbers of stop-codons or a frameshift. We therefore examined the ratio of accumulation of ORF-disrupting mutations per 100 AA in both groups of pseudogenes. A mutation rate of 0.28 was determined for the group of pseudogenes with one mutation and a significant higher rate of 1.21 (Mann-Whitney test; *P *= 8.052e - 7) was found for the set of pseudogenes with accumulated mutations.

Although transcribed pseudogenes have been experimentally identified [43], a significant part of pseudogenes are thought to be transcriptionally silent in comparison to protein-coding genes. We thus searched for sequence alignment with canine transcript sequences (Unigene april 08 [37]) to assess the transcription activity of the pseudogene predictions with two and more mutations. We obtained alignment for 14%, a result in agreement with a recent report [44] showing that 19% of pseudogenes are the sources of novel RNA transcripts. These data indicate that the predicted pseudogenes are mostly undetected as expressed sequences in comparison to gene predictions with intact ORF (53%) and therefore significantly correspond to untranscribed pseudogenes [44].

### Detecting nonfunctionality from *dN*/*dS *analysis

To assess independently of the presence of stop codons or frame-shifts, the validity of pseudogene predictions, we used the functionality test that uses the *d*_N_/*d*_S _ratio. Assuming a constant mutation rate, the *d*_N_/*d*_S _ratio between dog pseudogenes, for which a loss of function occurred, and their human functional orthologs should theoretically relax towards 0.57 (as the average of 1.0 in the absence of selection and 0.15 for negative selection as we calculated from the benchmark set) [10]. Thus, we calculated *d*_N_/*d*_S _ratio for each of the candidate pseudogene predictions in comparison to their human functional orthologous gene from pairwise transcripts pair alignments. For the pseudogene set with accumulated mutations, we calculated a median *d*_N_/*d*_S _of 0.50 indicating a considerable relaxation of selective constraints of the canine pseudogenes in comparison to their human functional orthologous (Figure 3 and Table 1). Furthermore, the *d*_N_/*d*_S _distributions obtained were shifted upwards in comparison to the benchmark set (Figure 4), which is significant to a Mann-Whitney test (*P *= 5.17e - 6), indicating relaxation of evolutionary constraints on the predicted pseudogenes. For the pseudogene set with one mutation, the median *d*_N_/*d*_S _of 0.18 was observed, suggesting no detectable differences in selective constraints between predicted pseudogenes from the canine sequence and their human functional counterparts. In addition, we analyzed whether the *d*_N_/*d*_S _ratio has an independent value before and after the stop codon among the predicted pseudogenes. In 26/28 instances, no significant differences were detected when comparing *d*_N_/*d*_S _ratio for the two parts of each gene. In two cases, the *d*_N_/*d*_S _value before the stop was indicative of strong selective constraints (<0.1), in comparison to the value detected after the stop (>0.9), which suggest that the biological function may have been preserved.

We next searched to determine if the canine predicted pseudogenes showed any deviations from the expected rate of evolution using a phylogenetic context that includes human and mouse gene sequences. Such variation in rate may reflect relaxation of constraints in the dog lineage. The deviation between dog predicted pseudogenes with multiple mutations and the human and mouse lineages differs clearly (*d*_N_/*d*_S _= 0.41 for dog, 0.19 for mouse and 0.26 for human; Kruskal-Wallis test: *P *= 1.04e - 2) while no significant deviation (*P *= 0.36) was observed for the set of pseudogenes with one mutation (Table 2). We therefore retained the 21 pseudogene predictions with both the higher *d*_N_/*d*_S _value as characterized by pairwise and phylogenetic approaches and high mutation rate as gene loss candidates.

**Table 2:** Evolutionary constraints (*dS *and *dN/d*S) for 1:1:1 orthologs among human, mouse and dog

*dN/dS *median	Predicted genes	Pseudogenes with several mutations	Pseudogenes with one mutation
Human	0.21	0.26	0.19

Dog	0.17	0.41	0.16

Mouse	0.15	0.19	0.13

### Gene loss identification

In addition to pseudogene identification, 11 gene predictions could not be detected with sufficient protein identity (average = 21.7%), both in the targeted genomic region (COIL) and in the whole canine sequence. For these predictions with no readily identifiable counterparts in dog, we searched for sequence alignment with canine expressed sequences (Unigene april 08) to address the underlying assumption that genes are not transcribed when placed in the context of highly degraded sequence. We identified sequence alignment in only three cases. These results showed that the gene predictions with poor sequence similarity were largely undetected as expressed sequences in comparison to gene predictions with intact ORF.

For the last subset of 49 canine genes that remained undetected in this study, we address the possibility that gene predictions could have been prevented because of a gap in the canine sequence assembly. We searched for gap content in the COILs that lack canine orthologous genes. For 12 COILs, the gap content was found to account for >10% of the total size of the COIL, seven-fold more than a random expectation set (n = 1000, gap = 1.32%) and manual inspection of sequence content resulted in identifying multiple sequence gaps. The 12 missing genes in those short targeted regions were therefore not retained in further analysis. Based on these results, a total of 37 undetected genes was considered and merged with the 11 gene predictions that could not be detected with sufficient protein identity and the 21 pseudogenes into a single set (n = 69) of gene loss candidates for further analyses (Figure 2 and Additional data file 5).

### Evolutionary scenarios of the canine gene losses

**Figure 5:**
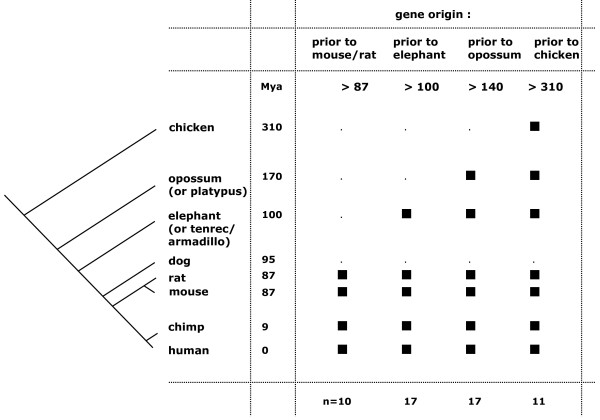
**Gene origin timing**. Timing of gene origin is assessed by determining the one-to-one orthologs between human and nine species listed on the left side of the figure. The species belong to Euarchontoglire (Primates and rodents), Xenarthra (Armadillo), Afrotheria (elephant and tenrec), Marsupial and Monotreme (opossum and platypus). Time of species divergence from the lineage leading to human is shown in MYA (million years ago). Filled squares represent the presence of the ortholog in the species. Numbers at the bottom of the figure denote the number of genes that display the presence/absence pattern across species.

Do we detect losses of genes that occur specifically in the dog or do such losses occur in other mammalian lineages as well? If so, do such losses correspond to the time the dog branch diverged from the Euarchontoglires (rodent/primate) lineage? One way to analyze these possibilities is to determine their phyletic pattern using ten species from chicken to human and to define the amount of time between gene origin and present. The timing of genes origin was defined by searching for 1:1 orthologs between human and nine species. In addition to human, chimp, mouse and rat genome sequence assemblies, we used scaffold assemblies of elephant, tenrec and armadillo from the Afrotheria and Xenarthra superorder and two non-placental genome assemblies of opossum and platypus. We also included the chicken sequence to infer gene origins that occurred as long as 310 million years ago (MYA) (Figure 5).

Orthologous genes were detected between human and all species (except dog) for 11 genes. Therefore, they have an origin that occurred before the separation of the mammals and birds lineages and have been functional for 310 million years (My). In addition, 17 genes were identified in all species of the opossum/platypus, elephant-tenrec-armadillo and Euarchontoglires branches, a period of 170 My, 17 in all species of the elephant-tenrec-armadillo and Euarchontoglires branches (100 My), and 10 in Euarchontoglires only (87 My) (Figure 5) [45].

Overall, 28 canine gene losses could be characterized as being functional in other species for more than 170 My and 10 genes were not detected before 87 My and therefore specifically arose in rodent and primate lineages. For these genes, postulating that they arose through duplication events of a parental gene, we searched for paralogs among all human genes. For seven genes (*ZNF426, WFDC12, ZIK1*, *HLA-SX-alpha*, *PNMA5*, *PNMA3, ZNF251*) we identified at least one paralog (sequence identity >30%) in the close vicinity of the parental gene (mean of 71 kb; range: 16–128 kb).

We further used the Ensembl reconciliation tree method [46] for checking possible duplication events specific of the primates and rodents lineages. Indeed, assuming that all homologous genes are known, the reconciliation of the gene tree with the species tree allows to distinguish duplication from speciation events and therefore orthologous from paralogous genes. Five genes (*ZNF426, ZIK1, HLA-SX-alpha, PNMA5, PNMA3*) have in-paralogs in the reference species suggesting a pattern of duplication event (Additional data file 6).

These results suggest that tandem duplication events have occurred and lead to specific in-paralogs in the branch leading to human species. Another contribution of this analysis is that it permits identification of 10 losses that occur in several lineages indicating multiple and independent gene loss events [47].

### Functional characteristics of gene losses

For the 28 canine-specific gene losses that have been functional for more than 170 My, we determined the functional annotation of the human genes using WebGestalt, a Web-based gene set analysis toolkit [48]. The classification using the GOTree sub-module includes seven genes that belong to the biological process of response to stimulus with *PROZ*, a vitamin K-dependent protein Z precursor involved in blood coagulation pathway and *SERPINA10 *a protein Z-dependant protease inhibitor that regulates factor Xa involved in blood coagulation. Moreover, it includes five genes involved in response to stimulus pathways that play a role in sensory function such as *UGT2A *which encodes an enzyme with transferase activity that may catalyze inactivation and facilitate elimination of odorants, *OR1Q1*, *OR1B1*, *ORN1 *which arethree olfactory receptors, and Noggin, a secreted polypeptide encoded by the *NOG *gene that appears to have pleiotropic effect, both early in development as well as in sensory perception of sound. Other genes of interest belong to families with at least six members such as *TBX22 *a transcription factor involved in the regulation of various aspects of embryonic development, in particular cell type specification and regulation of morphogenetic movements [49], and *MS4A3 *which is a subset of the superfamily of tetraspan transmembrane protein encoding genes. Several genes were classified with function involved in DNA repair, apoptosis and tumor formation such as *BOK *which encodes a Bcl-2 related protein and *PDE1B *which may play a role in apoptosis. To address the question of which tissue might be significantly affected by gene loss, we determined a gene-expression profile characterization per tissue based on the occurrence frequency of the ESTs profiles of human genes corresponding to the gene lost set using the tissue expression profile sub-module of WebGestalt. Testis-expressed gene expression profiles showed a significant over or under representation and, to a lesser extent, expression profiles related to placenta and kidney tissues did as well (Additional data file 7).

## Discussion

This study describes a multispecies comparative genomics approach that provides a methodology for improving genes prediction and detecting putative gene losses. When coupled to a strategy of phyletic pattern analysis, the approach allows differentiation of species-specific gene loss from multiple independent gene loss. Here, focusing on genes that were not detected in the whole-genome assembly of the dog but annotated in four rodents and primates species, we identified 232 new gene and we predicted 69 canine gene loss candidates of which 21 are identified as pseudogenes,

### Targeted gene prediction: strengths and limitations

A basic application of gene order-based approaches is the capacity to detect short conserved genomic context based on robust orthologous gene pair annotation. Therefore, results are limited by the source of gene annotation. In this study, we used the Ensembl annotation because of its good gene prediction coverage of the four species used as reference genomes. Since annotation of mammalian genome is a continuous process, our gene order-based approach may be improved over the course of time.

The use of short orthologous genomic intervals filtering has been well documented [28]. First, it reduces the cost of detecting false-positives as it filters out paralogs, with the exception of those caused by tandem gene duplication, and alignments to processed pseudogenes. Second, it allows a balance between sequence alignment sensitivity versus accuracy [50]. Alternatively, for more divergent sequences, alignment criteria may be relaxed in short pre-defined space where the background noise is significantly reduced compared to a genome scale search.

In our analysis, predictions may not provide an exhaustive list of gene predictions as inaccuracies may be generated by sequence artifacts that typically exist in draft sequence assemblies. Another issue related to prediction accuracy is the unexpected and unknown level of highly divergence at the nucleotide level. While scenarios of functional sequences with different evolutionary rate in different species exist [51], we postulated that using protein coding genes with a comparable evolutionary rate amongst four reference species reduces the possibility that a gene evolves independently in the dog species.

### Computational prediction of gene loss

A corollary to targeted gene prediction is that the absence of prediction strongly predicts gene relocation to a different region or chromosome or a gene loss event. Gene losses arise through retrotransposition or segmental or tandem duplications events followed by inactivation of one copy, or by degenerative mutations. We used a computational analysis to identify genes lost as pseudogenes based on various detrimental sequence mutations such as in-frame stop codons and frameshifts causing or resulting from loss of function. In this study, pseudogenes were separated in two groups, with the group of pseudogenes with one mutation (showing a low mutation rate) and the second group with an elevated mutation rate (>4 mutations, on average). Pseudogene predictions with one mutation could be overstated due to sequence artifacts that exist in the assembly. Indeed, stop codons and frameshifts are accommodated by algorithm like GeneWise. Other programs specifically designed for aligning pseudogenes such as GeneMapper [52] may be useful for addressing this problem. Another hypothesis is that pseudogene predictions have existed as pseudogenes (i.e. inactivated) for different amounts of time in the carnivore lineage. The formation of pseudogenes present in the canine genome could have been initiated by different or multiple events rather than have resulted from a continuous process over the course of time. Pseudogene characterization through the ratio of silent to replacement nucleotide substitution rates (*dN/dS*) may be a good indicator of changes in selective constraint that tend to be recent [53]. It is clear from our analysis that the *dN/dS *approach is useful to assess the evolutionary constraints that occur on nucleotide substitution. However, inferences of selection need to be treated with extreme caution.

### Functional impact of gene loss

We identified 28 gene losses that have been functional for more than 170 million years, a time period that extends from platypus to human (Figure 5). Losses of gene in a given species can be considered an adaptive event that may confer selective advantages to an organism [54]. Similarly to neutral losses, adaptive losses occurring ~95 MYA (for lineage leading to canid) are expected to leave genomic signatures with ORF-disrupting sequence mutations accumulation due to sequence degeneration. Here, the losses identified are based on ORF-disrupting sequence mutations, absence of EST validation and absence of significant similarity at the protein level. Although highly speculative, one hypothesis is that species-specific gene loss may confer a selective advantage in dog. Among the gene losses we identified were *PROZ*, a vitamin K-dependent protein Z precursor gene involved in response to stimulus that plays a role in blood coagulation. Mammalian blood coagulation is initiated and regulated by a complex network of interactions involved in normal hemostasis. Interestingly, Lindberg *et al*. describes a decrease of the expression of heme and globin related genes that correlate with the tameness trait in silver foxes suggesting that differences in behavior have a genetic basis [55]. A second hypothesis, is that gene loss may be a direct reflection of the loss of redundancy, where functionally overlapping genes cover for the loss of function as for genes involved in sensory functions [56,57].

## Conclusion

Among mammals, one-to-one orthologous correspondence can be defined for a large part of gene repertoires. Complex homologous relationships such as one-to-zero and many-to-many ones remain to be deciphered within gene families, for genes with divergent sequence as well as for species-specific genes that have emerged or have been lost through evolution. The combination of multispecies comparative genomics with in-depth gene prediction, accurate consideration of phylogenetic relationship, and timing of gene origin events can predict both gene structure and gene losses in newly sequenced genomes. This, in turn, enhances the integrity of reference genomes. The end result is a higher quality product for all sequenced genomes, regardless of the depth of sequence. We aim to see this approach applied to many other model organisms, thus enhancing the utility of the new sequencing resources throughout the comparative genomics community.

## Methods

### Gene datasets

Biomart [58] version 0.5 (Ensembl v.42) was used to collect orthologous protein-coding genes from the five genomes of interest: human (NCBI 36), chimp (Chimp 2.1), mouse (NCBI m36), rat (RGSC 3.4) and dog (CanFam 2.0). Ensembl Gene Id, orthologous relationships, locations in base pair for each species were downloaded and deposited into a MySQL database (v.4.1.12). The set of 412 protein-coding genes not annotated on the dog genome assembly with a 1:1:1:1:0 Human:Chimp:Mouse:Rat:Dog match was then extracted from the MySQL database.

### Synteny maps

We used the program AutoGRAPH [13] to construct pairwise synteny maps between reference genomes and tested genome. AutoGRAPH has been designed to construct synteny maps using genomic coordinates of ortholog pairs. The program transposes genomic coordinates into sequence of ordinal numbers and positions genes on an ordinal scale in relation to others on their respective chromosomes. Conserved segments ordered (CSO) can then be identified with respect to the ranking order. We only considered CSO containing a minimum of three genes. AutoGRAPH inferred the collinearity rate within CSO corresponding to the longest increasing gene order sequence between the two species divided by the total number of orthologs. We discarded CSO that had a collinearity rate less than 0.5. All synteny maps (n = 88) built in this work are presented in Additional data file 1 and can be downloaded.

### Gene structure prediction

The GeneWise program [6] (wise2-2-0) was used with default parameters to align each reference protein on the dog COIL forward and reverse strands (option -both) sequence. Predictions were post processed to pick up the highest genewise prediction, to compute sequence identity/similarity against reference proteins and to analyze splice sites conservation. Only predictions exhibiting at least 40% identity with reference proteins were retained. GeneWise was also used with the Hidden Markov model that uses HMM profiles generated with the HMMER package [59]. HMM-based prediction considers exons, introns and UTR regions as different states of gene structure that occupy subsequences of a sequence. A gene structure can be considered as an ordered set of state/sub-sequence pairs. A HMM-based prediction is considered as a predicted gene structure if probability of generating a gene structure is maximal over all possible states. Dynamic programming method for finding an optimal parse, or the best sequence of states has [10] been computed with the HMMER package.

### Homology searches

Reference transcript sequences were collated from Ensembl (v.42) and aligned against the canine sequence assembly (CanFam2) with the program Exonerate v1.2 [35]. Exonerate includes various models for aligning splice sites, combining speed and accuracy. We used the est2genome model, with a minimum perfect match of 18 bases to trigger alignments (dnawordlen 18). For each reference transcript, we retained the best five matching sequences.

Canine proteins inferred from the gene predictions were aligned against all canine transcripts with Exonerate using the coding2coding model. Canine predicted proteins were aligned on canine dbEST (est.fa 05/19/07 from UCSC) and UNIGENE (April 2008) using Exonerate with the protein2genome model.

The protein three-dimensional structure was available for 21 human genes. The sequences were retrieved via the Protein Data Bank. The amino sequences for the corresponding canine predictions were obtained from the genewise program prediction. Canine-human comparative modelling was determined using the SWISS-MODEL server [39]. Amino acid sequences are aligned between the primary structure of the human and the canine sequence. The three-dimensional model is constructed through the process implemented in the SWISS-MODEL server.

### *DN*/*dS *analysis

*DN*/*dS *analyses were conducted using the maximum-likelihood-based CODEML program (model = 0; PAML package) [60]. Sequence alignments of the whole coding region of the human orthologous sequence with canine prediction were realized with clustalW program. *Ds *values were calculated from pairwise alignments using all transcripts. To filter for possible inconsistencies among orthologous trancripts, we selected the transcript with the smallest phylogenetic distance using the smallest *dS*. For each dataset, we calculated a threshold on *dS *which two fold the median *dS*; all *dS *larger than this threshold were not used for the *dN*/*dS *calculation. *DN*/*dS *values of the benchmark set were extracted from Ensembl. *DN*/*dS *ratio in the phylogenetics context were calculated using CODEML program using the branch model set as model = 1 and run mode = 0. Sequence alignments of the whole coding region of the human, mouse and canine prediction orthologous sequence were realized with clustalW program

### Gene Ontology annotation

The Gene Ontology Tree Machine (GOTM) and WebGestalt programs [31,48] were used to retrieve GO term associated with ensembl gene ID. A hypergeometric test computes the statistical significance of overrepresentations of GO term compared to a reference complete list of genes. Only GO terms that were significantly over-represented (*P *< 1.0e - 3) were considered.

### Determining gene origin

For each of the 69 candidate gene losses, one-to-one orthologous gene was searched between human and nine species using the complete collection of orthologous protein-coding genes (Ensembl). Genome sequence assemblies were used for human, chimp, mouse, rat, monodelphis, platypus and chicken and scaffold assemblies for elephant, tenrec and armadillo. Timing of gene origin was inferred by determining the longest serie of one-to-one orthologs between the human and each of the nine species.

### P value calculation

We used the R package (R Development Core Team 2006. R: A language and environment for statistical computing. ) to test the statistical significance in comparing distinct distributions at each step of the method (Mann-Whitney, Kruskal-Wallis and Student's test).

## Abbreviations

ESTs: Expressed Sequence Tag; dbEST: database of EST; ORF: Open Reading Frame.

## Authors' contributions

TD prepared the data, carried out the comparative data analysis and contributed to the writing of the manuscript, JT worked on gene prediction analysis, AV carried out dN/dS analysis, CA participated in study design, EAO provided feedback throughput, suggested various analysis and worked on all drafts of the paper, FG participated in the data interpretation, and contributed to the writing of the manuscript, CH conceived of the study, participated in the data analysis and interpretation, and contributed to the writing of the manuscript. All authors read and approved the final manuscript.

## Supplementary Material

Additional file 1 **Human-dog synteny map: Example of human chromosome 5.** An example of the synteny map built between human chromosome 5 and the dog genome. Click here for file

Additional file 2 **Synteny maps characteristics.** The data indicates the main characteristics of the synteny maps. Click here for file

Additional file 3 **Characterization of Consensus Orthologous IntervaLs (COILs) containing missing genes.** These data file lists the characteristics of the Consensus Orthologous Intervals. Click here for file

Additional file 4 **List of the 232 new predicted canine genes.** This table lists the 232 new gene predictions using the human gene identifiers from Ensembl. Click here for file

Additional file 5 **List of the 69 candidate gene losses.** This table lists the gene losses using the human gene identifiers from Ensembl. Click here for file

Additional file 6 **Gene/species tree reconcilation.** These data provide the gene/species tree reconcilation that show the possible duplication events specific of the primates and rodents lineages. Click here for file

Additional file 7 **Gene-expression profile characterization per tissue with significant over and under representation.** The data provided show gene-expression profile characterization per tissue. Click here for file
