# Supplementary material for: Revisiting the missing protein-coding gene catalog of the domestic dog
Source: BMC Genomics. 2009 Feb 4;10:62. doi: 10.1186/1471-2164-10-62 (PMC2644713; doi:10.1186/1471-2164-10-62)
Supplement: Additional file 1 — Human-dog synteny map: Example of human chromosome 5. An example of the synteny map built between human chromosome 5 and the dog genome. [file 1471-2164-10-62-S1.pdf]

Additional data file 1 : Human-dog synteny map : Example of human chromosome 5

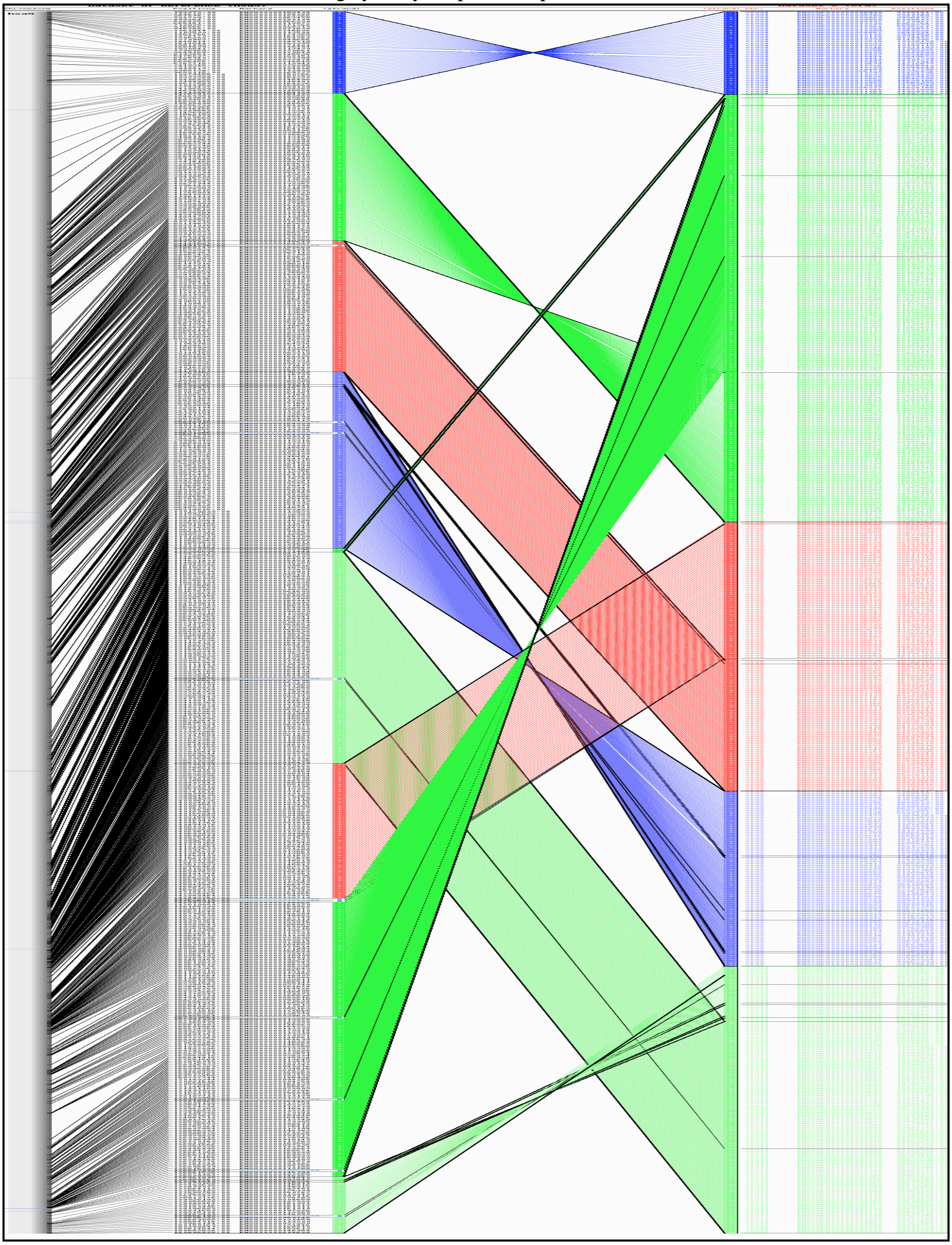

Chromosome 5 from human, the reference species, is shown on the left hand side of the figure. The right part corresponds to the canine genome. For both species, genes are identified by their ID and genomic coordinates. The colored lines connecting orthologous genes identify CS and CSO and show the conservation of gene order. Black lines between colored segments represent breakpoints between CS and/or CSO. All synteny maps; 23 for human-dog; 24 chimp-dog; 20 mouse-dog and 21 rat-dog are available as supplemental data at : http://genoweb.univ-rennes1.fr/tom_dog/Supplementary/
