# Supplementary material for: Revisiting the missing protein-coding gene catalog of the domestic dog
Source: BMC Genomics. 2009 Feb 4;10:62. doi: 10.1186/1471-2164-10-62 (PMC2644713; doi:10.1186/1471-2164-10-62)
Supplement: Additional file 2 — Synteny maps characteristics. The data indicates the main characteristics of the synteny maps. [file 1471-2164-10-62-S2.pdf]

Additional data file 2 :

Synteny maps characteristics

synteny maps	1:1 orthologs	Conserved Segment Ordered (CSO)	CSO mean size (Mb)	gene-order conservation rate
Human-Dog	14,997	218	12.58	95.6%
Chimp-Fog	14,798	229	11.93	94.8%
Mouse-Dog	14,667	326	7.19	95.7%
Rat-Dog	14,065	325	7.47	94.6%

Synteny maps are constructed using 1:1 orthologs as anchors. Conserved Segments Ordered (CSO) are identified with respect to the ranking order. We only considered CSO containing a minimum of three genes.
