# Supplementary material for: Revisiting the missing protein-coding gene catalog of the domestic dog
Source: BMC Genomics. 2009 Feb 4;10:62. doi: 10.1186/1471-2164-10-62 (PMC2644713; doi:10.1186/1471-2164-10-62)
Supplement: Additional file 3 — Characterization of Consensus Orthologous IntervaLs (COILs) containing missing genes. These data file lists the characteristics of the Consensus Orthologous Intervals. [file 1471-2164-10-62-S3.pdf]

Additional data file 3:

Characterization of Consensus Orthologous IntervaLs (COILs) containing missing genes

	# of gene	% of gene localized in telomeric region	COIL mean size (kb)	COIL gap content (%)	COIL repeat content (%)	COIL GC content (%)
missing genes	383	44.4	347	2.23	37.33	46.23
random set	1000	31.0	375	1.32	35.88	45.63
