# Supplementary material for: Revisiting the missing protein-coding gene catalog of the domestic dog
Source: BMC Genomics. 2009 Feb 4;10:62. doi: 10.1186/1471-2164-10-62 (PMC2644713; doi:10.1186/1471-2164-10-62)
Supplement: Additional file 4 — List of the 232 new predicted canine genes. This table lists the 232 new gene predictions using the human gene identifiers from Ensembl. [file 1471-2164-10-62-S4.pdf]

Additional data file 4.

List of gene predictions :

Canine Gene_predictions (human gene Id.)
ENSG00000017621
ENSG00000044524
ENSG00000049540
ENSG00000059377
ENSG00000062370
ENSG00000064199
ENSG00000070031
ENSG00000076984
ENSG00000083093
ENSG00000083838
ENSG00000083842
ENSG00000085644
ENSG00000086289
ENSG00000087250
ENSG00000087586
ENSG00000089505
ENSG00000093134
ENSG00000099800
ENSG00000099972
ENSG00000101210
ENSG00000101448
ENSG00000102119
ENSG00000104472
ENSG00000104886
ENSG00000104921
ENSG00000105499
ENSG00000105583
ENSG00000105967
ENSG00000106261
ENSG00000106631
ENSG00000108947
ENSG00000110876
ENSG00000111358
ENSG00000112110
ENSG00000116819
ENSG00000121743
ENSG00000122068
ENSG00000123200
ENSG00000123689
ENSG00000124812
ENSG00000125285
ENSG00000125356
ENSG00000125531
ENSG00000125823
ENSG00000125846

ENSG00000126264
ENSG00000126545
ENSG00000128645
ENSG00000129235
ENSG00000129451
ENSG00000130037
ENSG00000130193
ENSG00000130640
ENSG00000130943
ENSG00000132603
ENSG00000134326
ENSG00000134986
ENSG00000135547
ENSG00000135643
ENSG00000136352
ENSG00000136521
ENSG00000136539
ENSG00000136866
ENSG00000137098
ENSG00000137338
ENSG00000137414
ENSG00000137720
ENSG00000139187
ENSG00000139826
ENSG00000140015
ENSG00000141040
ENSG00000141255
ENSG00000142856
ENSG00000144821
ENSG00000145242
ENSG00000145685
ENSG00000145839
ENSG00000145850
ENSG00000150526
ENSG00000151079
ENSG00000152592
ENSG00000153498
ENSG00000154548
ENSG00000154589
ENSG00000154768
ENSG00000154864
ENSG00000155254
ENSG00000155640
ENSG00000155858
ENSG00000156097
ENSG00000156853
ENSG00000157502
ENSG00000158428
ENSG00000158691
ENSG00000158716

ENSG00000159596
ENSG00000160345
ENSG00000161298
ENSG00000161958
ENSG00000162510
ENSG00000162585
ENSG00000162817
ENSG00000163362
ENSG00000163467
ENSG00000165370
ENSG00000165828
ENSG00000165973
ENSG00000166529
ENSG00000166619
ENSG00000166704
ENSG00000166788
ENSG00000166987
ENSG00000167100
ENSG00000167377
ENSG00000167384
ENSG00000167617
ENSG00000167641
ENSG00000168282
ENSG00000168496
ENSG00000168875
ENSG00000169131
ENSG00000169509
ENSG00000169740
ENSG00000169860
ENSG00000169926
ENSG00000169957
ENSG00000169976
ENSG00000170382
ENSG00000170677
ENSG00000170748
ENSG00000170855
ENSG00000171425
ENSG00000171443
ENSG00000171476
ENSG00000171695
ENSG00000171786
ENSG00000172209
ENSG00000172466
ENSG00000173369
ENSG00000173699
ENSG00000173875
ENSG00000175520
ENSG00000175646
ENSG00000176584
ENSG00000176641

ENSG00000176774
ENSG00000176788
ENSG00000176925
ENSG00000177202
ENSG00000177414
ENSG00000177669
ENSG00000177700
ENSG00000177932
ENSG00000178042
ENSG00000178187
ENSG00000178235
ENSG00000178257
ENSG00000178338
ENSG00000178394
ENSG00000178690
ENSG00000178732
ENSG00000179165
ENSG00000179673
ENSG00000180035
ENSG00000180543
ENSG00000180697
ENSG00000180834
ENSG00000181467
ENSG00000181617
ENSG00000181656
ENSG00000181894
ENSG00000182631
ENSG00000182791
ENSG00000183103
ENSG00000183134
ENSG00000183150
ENSG00000183161
ENSG00000183484
ENSG00000183495
ENSG00000183729
ENSG00000183773
ENSG00000183971
ENSG00000183979
ENSG00000184343
ENSG00000184486
ENSG00000184682
ENSG00000184898
ENSG00000185056
ENSG00000185155
ENSG00000185271
ENSG00000185689
ENSG00000185842
ENSG00000185863
ENSG00000185942
ENSG00000186452

ENSG00000187815
ENSG00000188243
ENSG00000188691
ENSG00000188803
ENSG00000188959
ENSG00000189266
ENSG00000189420
ENSG00000196150
ENSG00000196196
ENSG00000196867
ENSG00000197062
ENSG00000197083
ENSG00000197261
ENSG00000197487
ENSG00000198088
ENSG00000198440
ENSG00000198612
ENSG00000198680
ENSG00000198821
ENSG00000203667
ENSG00000203727
ENSG00000203733
ENSG00000203778
ENSG00000203910
ENSG00000203963
ENSG00000204006
ENSG00000204193
ENSG00000204222
ENSG00000204300
ENSG00000204856
ENSG00000204954
ENSG00000204978
ENSG00000205002
ENSG00000205301
ENSG00000205423
ENSG00000205476
ENSG00000205707
