# Supplementary material for: Revisiting the missing protein-coding gene catalog of the domestic dog
Source: BMC Genomics. 2009 Feb 4;10:62. doi: 10.1186/1471-2164-10-62 (PMC2644713; doi:10.1186/1471-2164-10-62)
Supplement: Additional file 5 — List of the 69 candidate gene losses. This table lists the gene losses using the human gene identifiers from Ensembl. [file 1471-2164-10-62-S5.pdf]

**Additional data file 5.**

**List of gene losses :**

| *Gene_loss* |
|---|
| ENSG00000025708 |
| ENSG00000050628 |
| ENSG00000149609 |
| ENSG00000152463 |
| ENSG00000172689 |
| ENSG00000177291 |
| ENSG00000178015 |
| ENSG00000180044 |
| ENSG00000185245 |
| ENSG00000186143 |
| ENSG00000188112 |
| ENSG00000062524 |
| ENSG00000091106 |
| ENSG00000112238 |
| ENSG00000124143 |
| ENSG00000126856 |
| ENSG00000137497 |
| ENSG00000145555 |
| ENSG00000153029 |
| ENSG00000159648 |
| ENSG00000174930 |
| ENSG00000178342 |
| ENSG00000179088 |
| ENSG00000182318 |
| ENSG00000183691 |
| ENSG00000184900 |
| ENSG00000186047 |
| ENSG00000197651 |
| ENSG00000198883 |
| ENSG00000203734 |
| ENSG00000204851 |
| ENSG00000205184 |
| ENSG00000083817 |
| ENSG00000122145 |
| ENSG00000123360 |
| ENSG00000126231 |
| ENSG00000127528 |
| ENSG00000130818 |
| ENSG00000132846 |
| ENSG00000140093 |
| ENSG00000145014 |
| ENSG00000149516 |
| ENSG00000154274 |
| ENSG00000157111 |
| ENSG00000163810 |

| |
|---|
| ENSG00000165202 |
| ENSG00000167644 |
| ENSG00000168703 |
| ENSG00000169327 |
| ENSG00000171484 |
| ENSG00000171501 |
| ENSG00000171649 |
| ENSG00000172899 |
| ENSG00000173464 |
| ENSG00000173610 |
| ENSG00000173954 |
| ENSG00000176381 |
| ENSG00000176720 |
| ENSG00000179859 |
| ENSG00000183837 |
| ENSG00000197721 |
| ENSG00000198169 |
| ENSG00000198185 |
| ENSG00000198261 |
| ENSG00000203760 |
| ENSG00000204614 |
| ENSG00000204616 |
| ENSG00000204671 |
| ENSG00000205795 |
