# Supplementary material for: Revisiting the missing protein-coding gene catalog of the domestic dog
Source: BMC Genomics. 2009 Feb 4;10:62. doi: 10.1186/1471-2164-10-62 (PMC2644713; doi:10.1186/1471-2164-10-62)

ZNF426 (ENSG00000130818) :

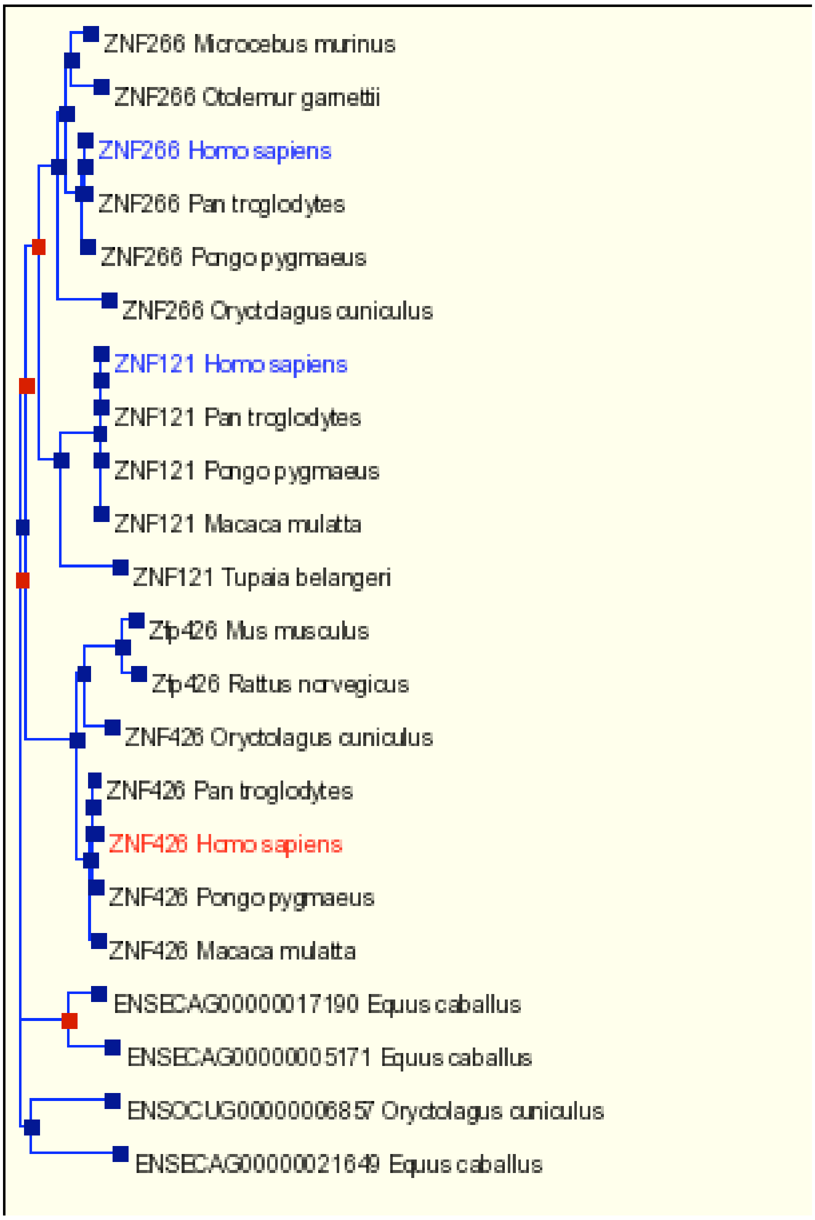

Legend :

Gene ID Species A current gene
Gene ID Species A within-sp. paralogue

speciation node
duplication node

ZIK1 (ENSG00000171649) :

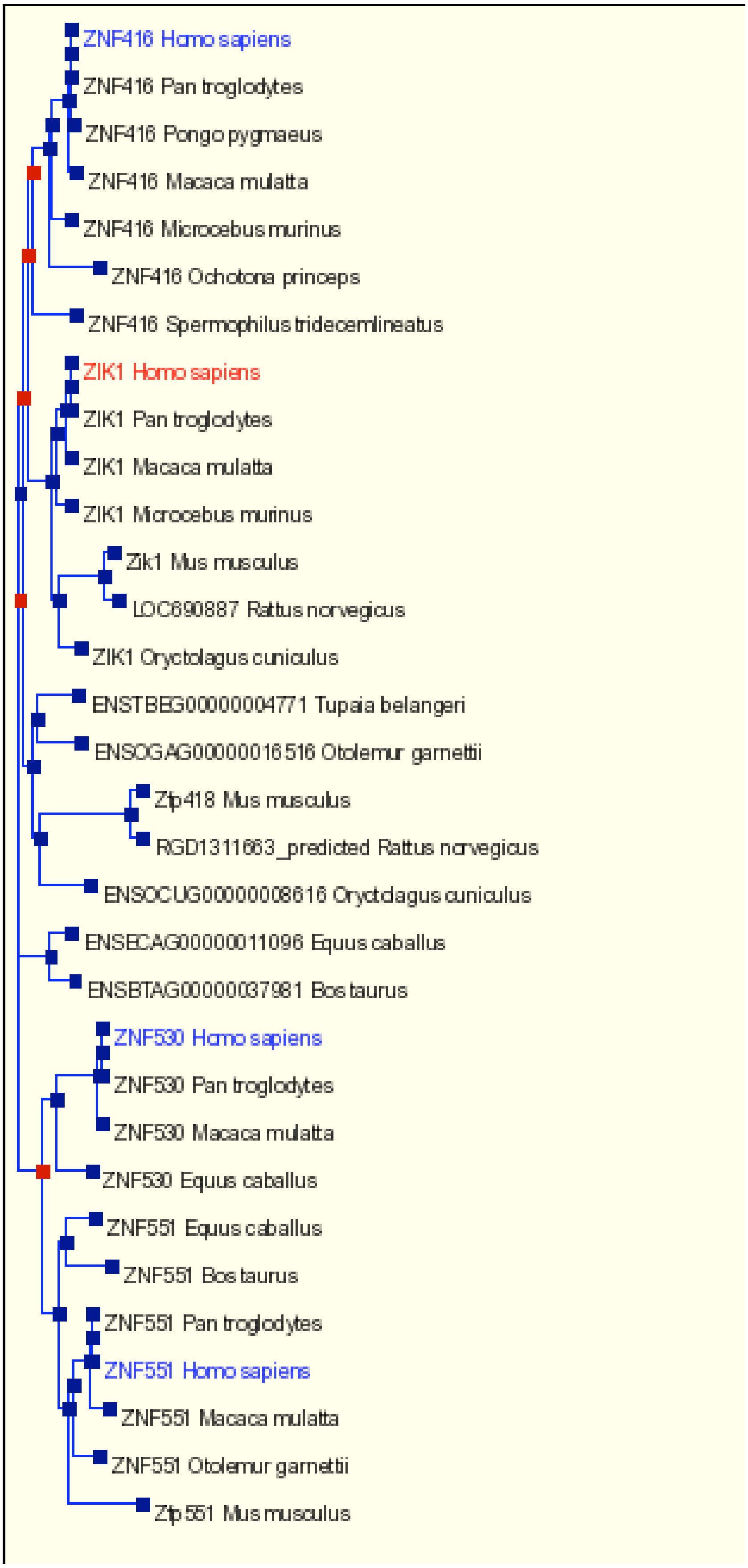

PNMA5 (ENSG00000198883) :

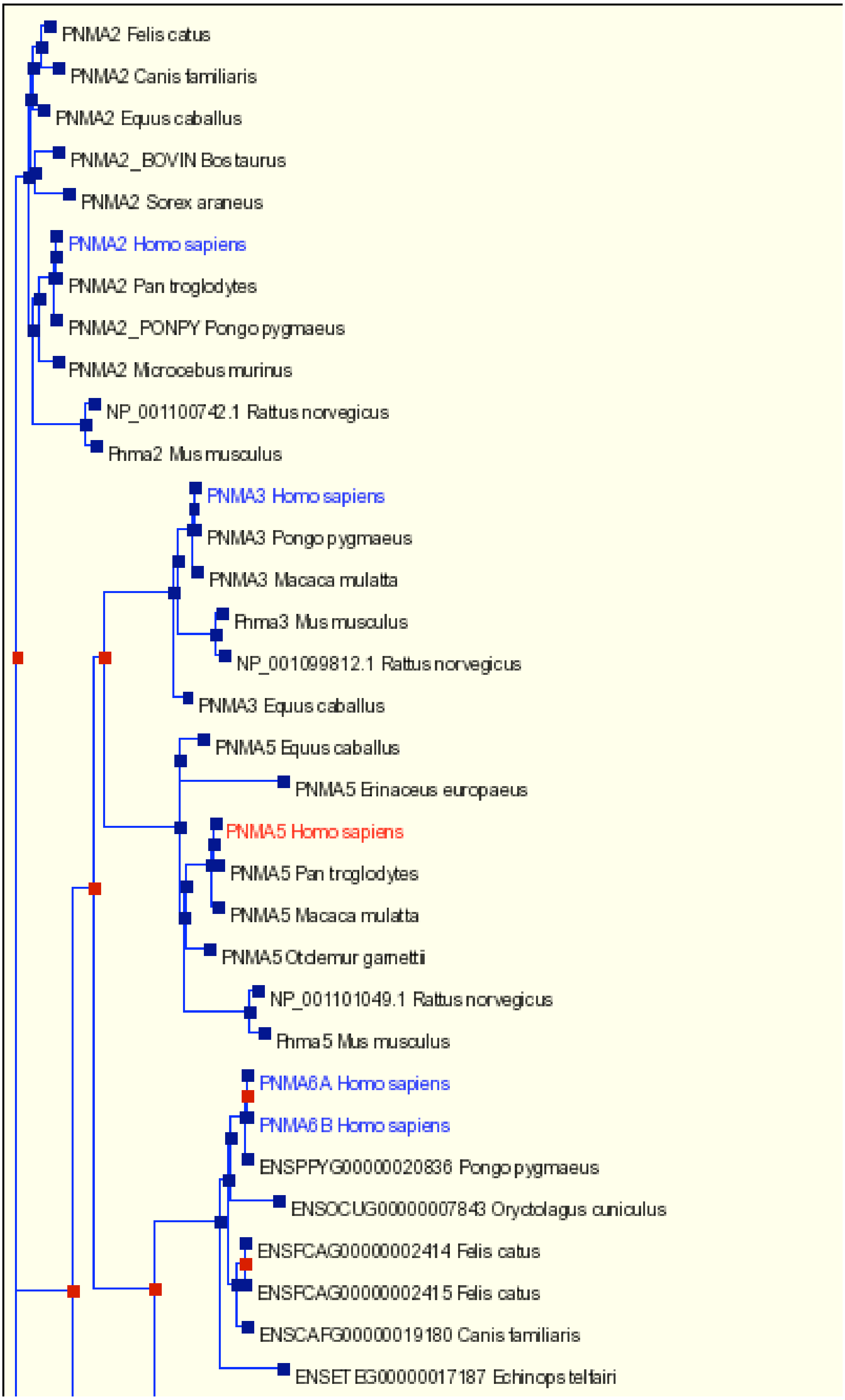

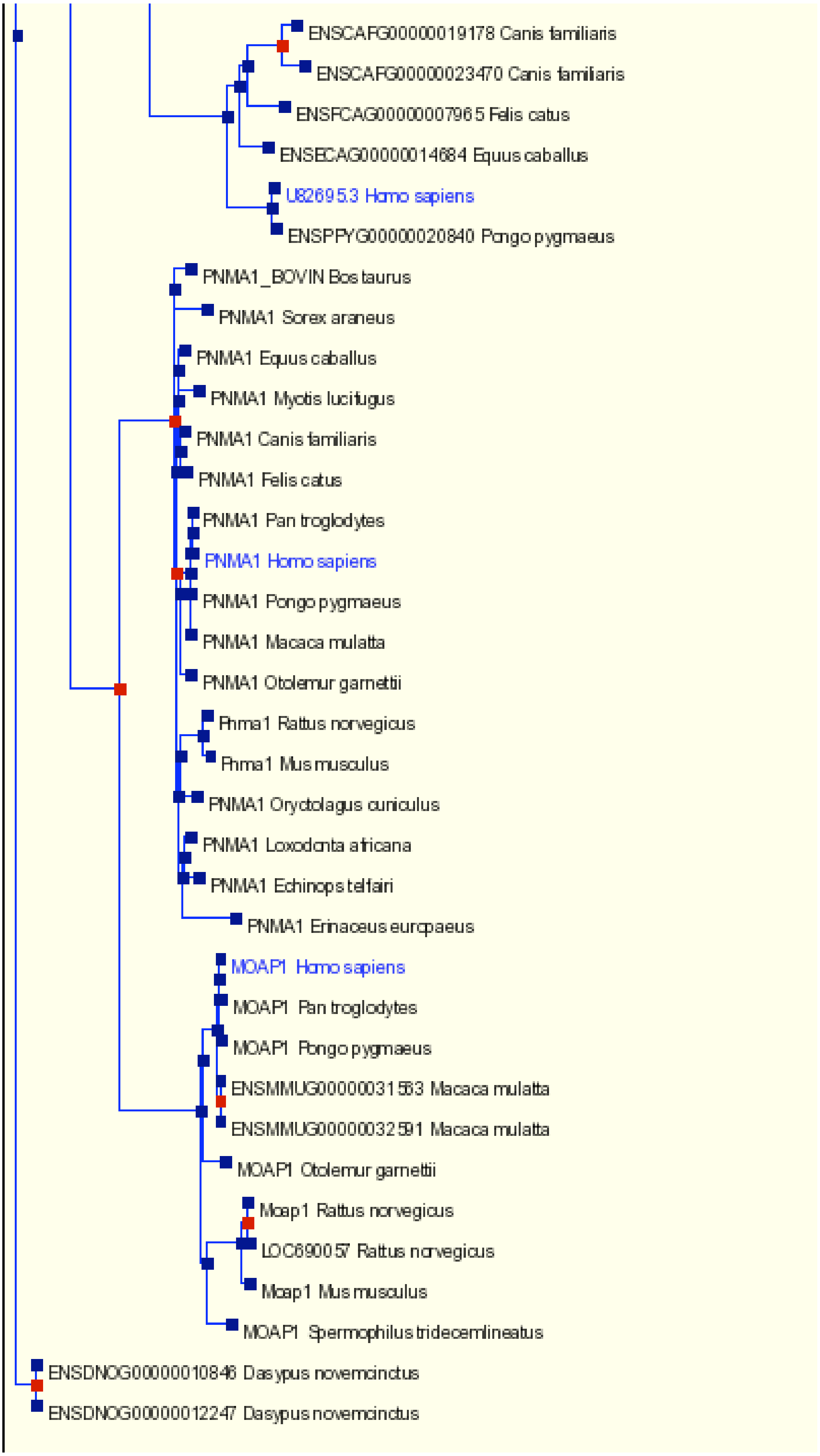

ENSCAFG00000019178 Canis familiaris
ENSCAFG00000023470 Canis familiaris
ENSFCAG00000007965 Felis catus
ENSECAG00000014684 Equus caballus
U82695.3 Homo sapiens
ENSPPYG00000020840 Pongo pygmaeus
PNMA1_BOVIN Bos taurus
PNMA1 Sorex araneus
PNMA1 Equus caballus
PNMA1 Myotis lucifugus
PNMA1 Canis familiaris
PNMA1 Felis catus
PNMA1 Pan troglodytes
PNMA1 Homo sapiens
PNMA1 Pongo pygmaeus
PNMA1 Macaca mulatta
PNMA1 Otolemur garnettii
Pnma1 Rattus norvegicus
Pnma1 Mus musculus
PNMA1 Oryctolagus cuniculus
PNMA1 Loxodonta africana
PNMA1 Echinops telfairi
PNMA1 Erinaceus europaeus
MOAP1 Homo sapiens
MOAP1 Pan troglodytes
MOAP1 Pongo pygmaeus
ENSMMUG00000031563 Macaca mulatta
ENSMMUG00000032591 Macaca mulatta
MOAP1 Otolemur garnettii
Moap1 Rattus norvegicus
LOC690057 Rattus norvegicus
Moap1 Mus musculus
MOAP1 Spermophilus tridecemlineatus
ENSDNOG00000010846 Dasypus novemcinctus
ENSDNOG00000012247 Dasypus novemcinctus

PNMA3 (ENSG00000183837) :

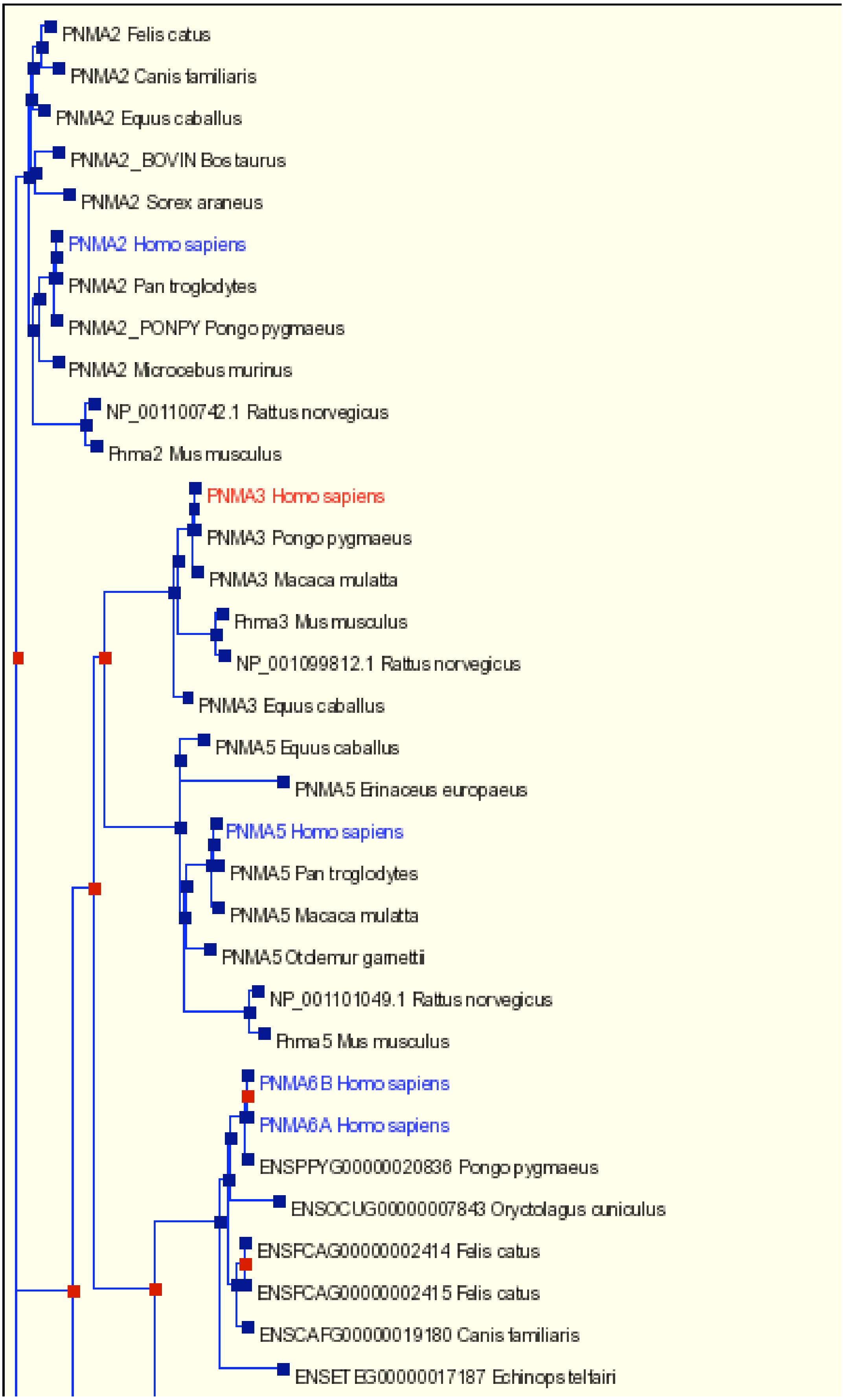

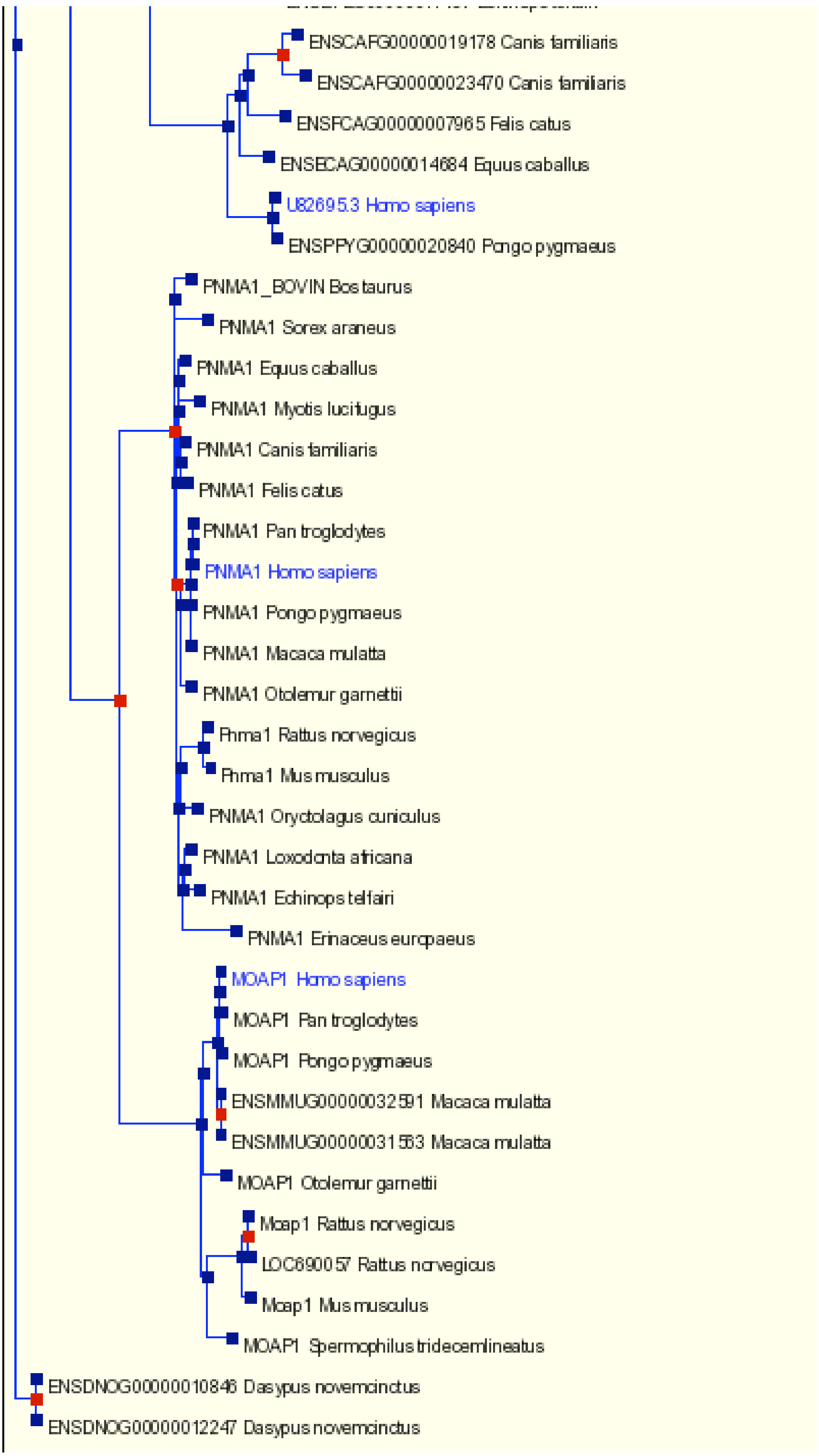
ENSCAFG00000019178 Canis familiaris
ENSCAFG00000023470 Canis familiaris
ENSFCAG00000007965 Felis catus
ENSECAG00000014684 Equus caballus
U82695.3 Homo sapiens
ENSPPYG00000020840 Pongo pygmaeus
PNMA1_BOVIN Bos taurus
PNMA1 Sorex araneus
PNMA1 Equus caballus
PNMA1 Myotis lucifugus
PNMA1 Canis familiaris
PNMA1 Felis catus
PNMA1 Pan troglodytes
PNMA1 Homo sapiens
PNMA1 Pongo pygmaeus
PNMA1 Macaca mulatta
PNMA1 Otolemur garnettii
Pnma1 Rattus norvegicus
Pnma1 Mus musculus
PNMA1 Oryctolagus cuniculus
PNMA1 Loxodonta africana
PNMA1 Echinops telfairi
PNMA1 Erinaceus europaeus
MOAP1 Homo sapiens
MOAP1 Pan troglodytes
MOAP1 Pongo pygmaeus
ENSMMUG00000032591 Macaca mulatta
ENSMMUG00000031553 Macaca mulatta
MOAP1 Otolemur garnettii
Moap1 Rattus norvegicus
LOC690057 Rattus norvegicus
Moap1 Mus musculus
MOAP1 Spermophilus tridecemlineatus
ENSDNOG00000010846 Dasypus novemcinctus
ENSDNOG00000012247 Dasypus novemcinctus

AL645931 (ENSG00000172899) :

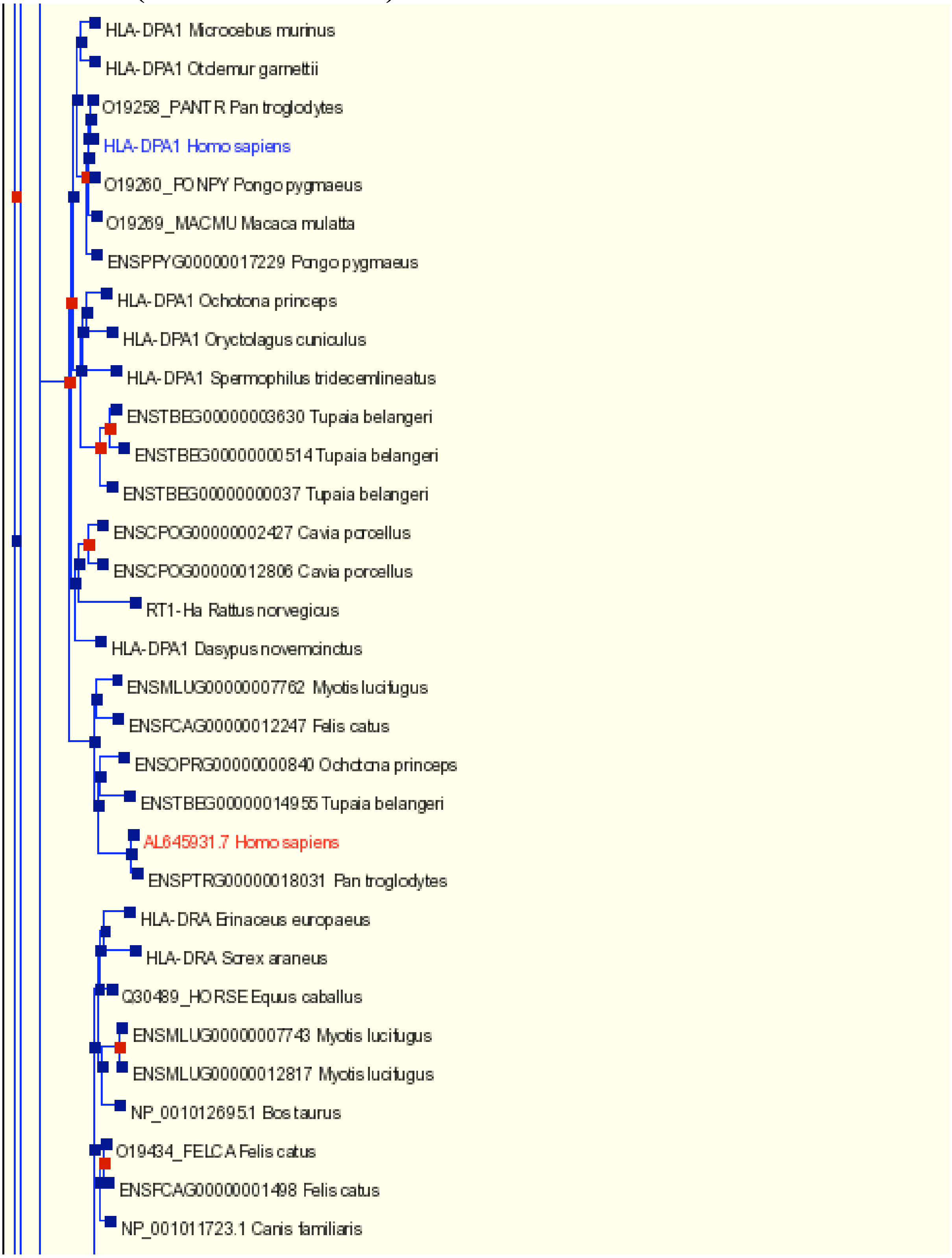

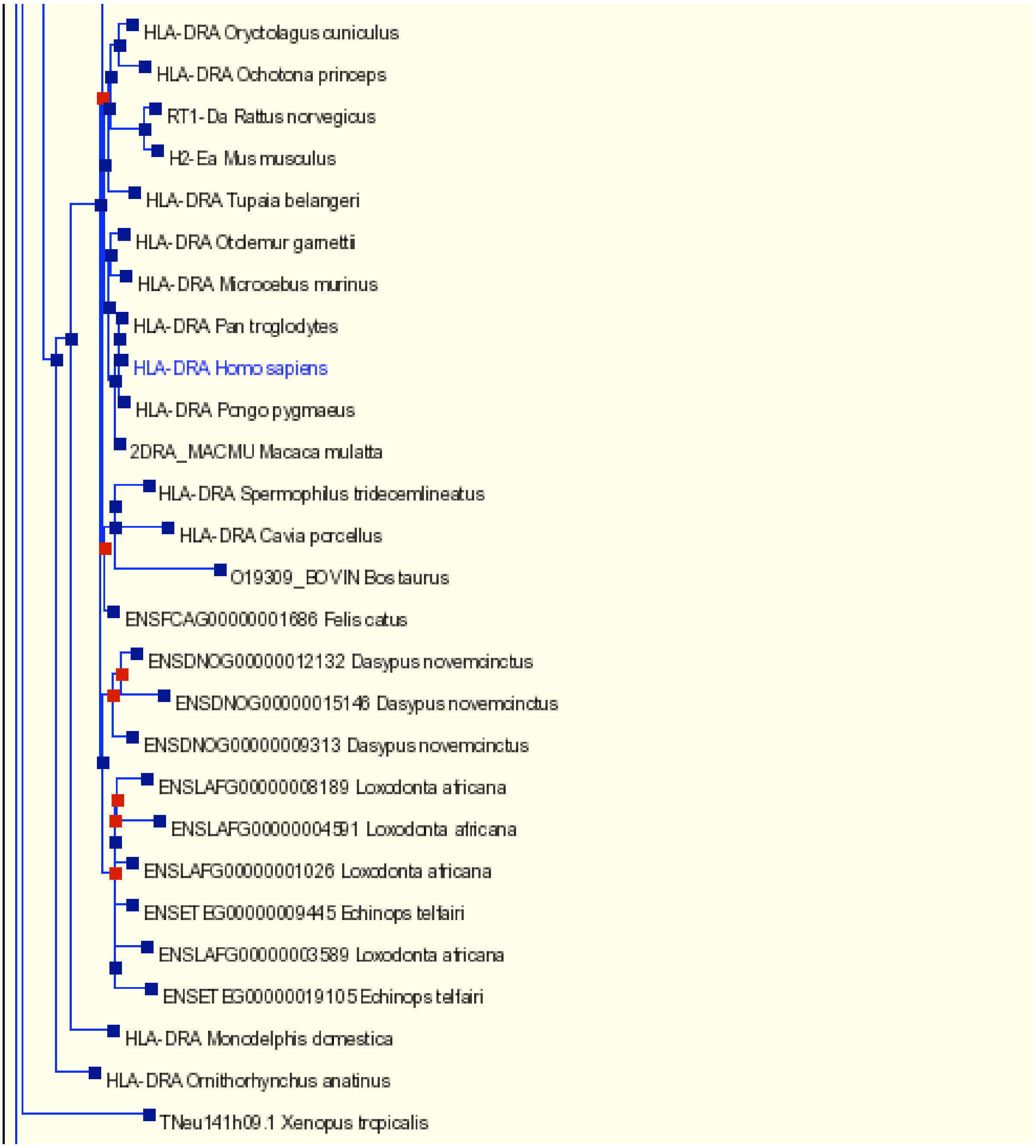
HLA-DRA Oryctolagus cuniculus
HLA-DRA Ochotona princeps
RT1-Da Rattus norvegicus
H2-Ea Mus musculus
HLA-DRA Tupaia belangeri
HLA-DRA Otolemur garnettii
HLA-DRA Microcebus murinus
HLA-DRA Pan troglodytes
HLA-DRA Homo sapiens
HLA-DRA Pongo pygmaeus
2DRA_MACMU Macaca mulatta
HLA-DRA Spermophilus tridecemlineatus
HLA-DRA Cavia porcellus
O19309_BOVIN Bos taurus
ENSFCAG00000001686 Felis catus
ENSDNOG00000012132 Dasypus novemcinctus
ENSDNOG00000015146 Dasypus novemcinctus
ENSDNOG00000009313 Dasypus novemcinctus
ENSLAFG00000008189 Loxodonta africana
ENSLAFG00000004591 Loxodonta africana
ENSLAFG00000001026 Loxodonta africana
ENSETEG00000009445 Echinops telfairi
ENSLAFG00000003589 Loxodonta africana
ENSETEG00000019105 Echinops telfairi
HLA-DRA Monodelphis domestica
HLA-DRA Ornithorhynchus anatinus
TNeu141h09.1 Xenopus tropicalis

CYS1 (ENSG00000205795) :

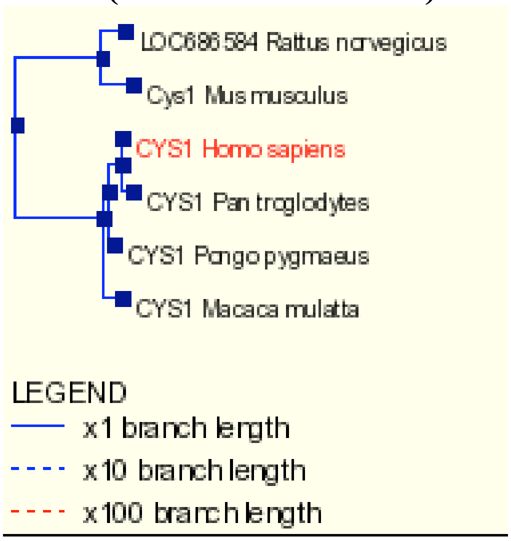

ZNF251 (ENSG00000198169) :

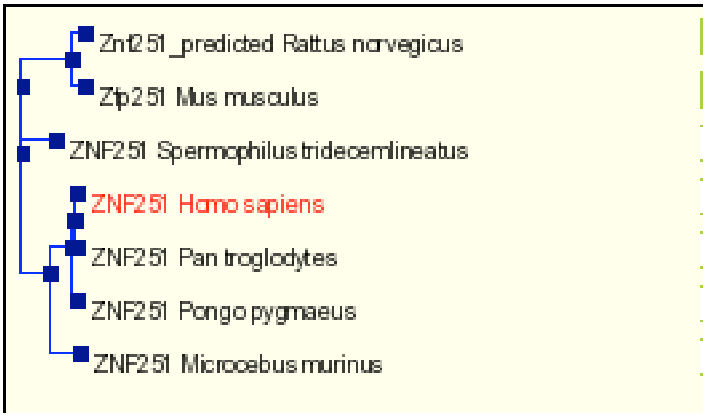

WFDC12 (ENSG00000168703) :

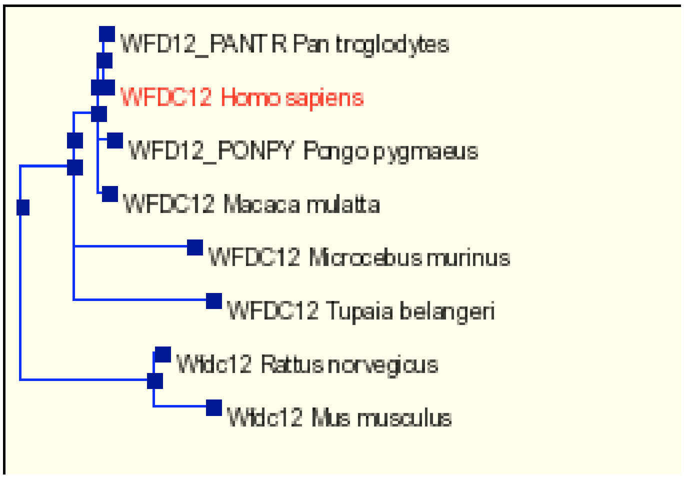

GP1BA (ENSG00000185245) :

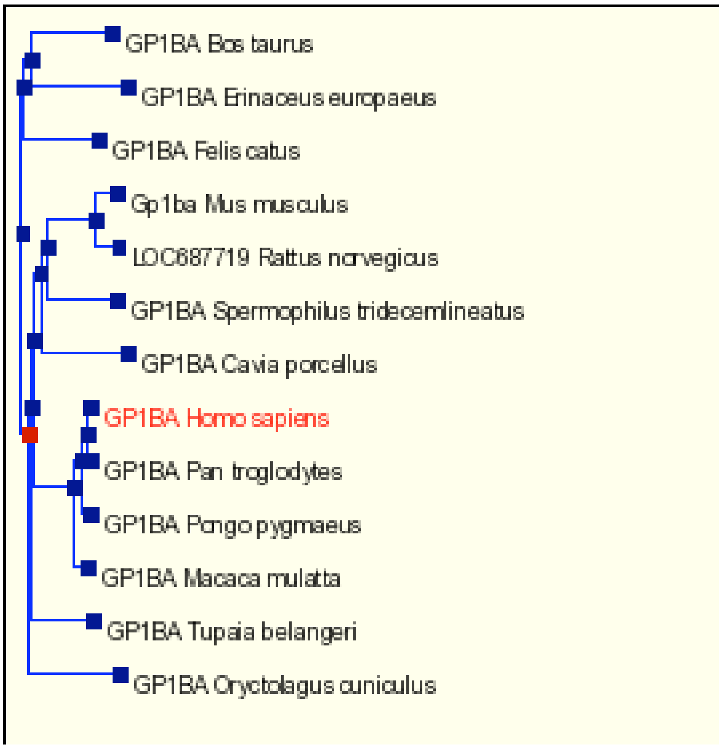

MYO10 (ENSG00000145555) :

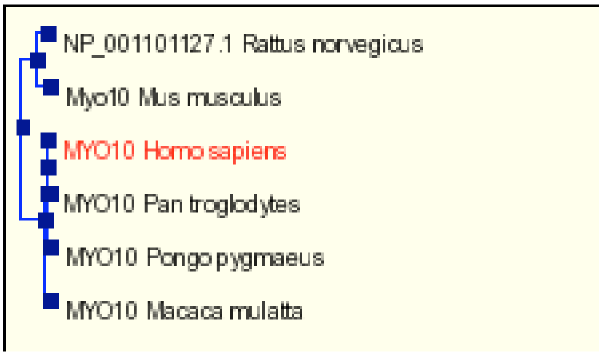

Supplement: Additional file 6 — Gene/species tree reconcilation. These data provide the gene/species tree reconcilation that show the possible duplication events specific of the primates and rodents lineages. [file 1471-2164-10-62-S6.pdf]
