# Supplementary material for: Revisiting the missing protein-coding gene catalog of the domestic dog
Source: BMC Genomics. 2009 Feb 4;10:62. doi: 10.1186/1471-2164-10-62 (PMC2644713; doi:10.1186/1471-2164-10-62)
Supplement: Additional file 7 — Gene-expression profile characterization per tissue with significant over and under representation. The data provided show gene-expression profile characterization per tissue. [file 1471-2164-10-62-S7.pdf]

Additional data file 6: Human gene-expression profile characterization per tissue with significant over (red) and under (blue) representation

testis

Input ID	*Symbol*	*Name*	**Over/Under representation**
ENSG00000149609	C20orf144	chromosome 20 open reading frame 144	2.450e-09
ENSG00000122145	TBX22	T-box 22	2.654e-08
ENSG00000152463	OLAH	oleoyl-ACP hydrolase	6.544e-06
ENSG00000159648			1.992e-05
ENSG00000176720	BOK	BCL2-related ovarian killer	-1.030e-03
ENSG00000137497	NUMA1	nuclear mitotic apparatus protein 1	-6.864e-03
ENSG00000123360	PDE1B	phosphodiesterase 1B, calmodulin-dependent	-1.135e-02
ENSG00000178015	GPR150	G protein-coupled receptor 150	4.872e-02
ENSG00000184900	SUMO3	SMT3 suppressor of mif two 3 homolog 3 (S. cerevisiae)	9.086e-02
ENSG00000154274	C4orf19	chromosome 4 open reading frame 19	-1.720e-01
ENSG00000145014	TMEM44	transmembrane protein 44	-4.087e-01
ENSG00000140093	SERPINA10	serpin peptidase inhibitor, clade A (alpha-1 antiproteinase, antitrypsin), member 10	-4.376e-01
ENSG00000153029	MR1	major histocompatibility complex, class I-related	5.781e-01

kidney

Input ID	*Symbol*	*Name*	**Over/Under representation**
ENSG00000173610	UGT2A1	UDP glucuronosyltransferase 2 family, polypeptide A1	9.938e-06
ENSG00000126231	PROZ	protein Z, vitamin K-dependent plasma glycoprotein	7.731e-05
ENSG00000137497	NUMA1	nuclear mitotic apparatus protein 1	-4.014e-04
ENSG00000157111	TMEM171	transmembrane protein 171	7.460e-04

ENSG00000153029	MR1	major histocompatibility complex, class I-related	2.090e-03
ENSG00000154274	C4orf19	chromosome 4 open reading frame 19	1.130e-02
ENSG00000176720	BOK	BCL2-related ovarian killer	7.222e-02
ENSG00000123360	PDE1B	phosphodiesterase 1B, calmodulin-dependent	-9.801e-02
ENSG00000152463	OLAH	oleoyl-ACP hydrolase	-1.497e-01
ENSG00000184900	SUMO3	SMT3 suppressor of mif two 3 homolog 3 (S. cerevisiae)	-3.119e-01
ENSG00000145014	TMEM44	transmembrane protein 44	-4.737e-01

Placenta

Input ID	*Symbol*	*Name*	**Over/Under representation**
ENSG00000154274	C4orf19	chromosome 4 open reading frame 19	1.024e-24
ENSG00000204614	TRIM40	tripartite motif-containing 40	4.862e-17
ENSG00000152463	OLAH	oleoyl-ACP hydrolase	4.751e-09
ENSG00000137497	NUMA1	nuclear mitotic apparatus protein 1	-1.449e-04
ENSG00000153029	MR1	major histocompatibility complex, class I-related	3.427e-03
ENSG00000176720	BOK	BCL2-related ovarian killer	-4.179e-03
ENSG00000149609	C20orf144	chromosome 20 open reading frame 144	1.592e-02
ENSG00000145014	TMEM44	transmembrane protein 44	-9.665e-02
ENSG00000183691	NOG	noggin	2.779e-01
ENSG00000184900	SUMO3	SMT3 suppressor of mif two 3 homolog 3 (S. cerevisiae)	-4.083e-01

Colon

Input ID	*Symbol*	*Name*	**Over/Under representation**
ENSG00000137497	NUMA1	nuclear mitotic apparatus protein 1	3.507e-18
ENSG00000154274	C4orf19	chromosome 4 open reading frame 19	2.780e-03
ENSG00000157111	TMEM171	transmembrane protein 171	3.335e-02
ENSG00000123360	PDE1B	phosphodiesterase 1B, calmodulin-dependent	-1.154e-01
ENSG00000204614	TRIM40	tripartite motif-containing 40	1.349e-01
ENSG00000152463	OLAH	oleoyl-ACP hydrolase	-1.716e-01
ENSG00000184900	SUMO3	SMT3 suppressor of mif two 3 homolog 3 (S. cerevisiae)	-2.861e-01

ENSG00000145014	TMEM44	transmembrane protein 44	-2.998e-01
ENSG00000176720	BOK	BCL2-related ovarian killer	4.450e-01
ENSG00000153029	MR1	major histocompatibility complex, class I-related	-4.823e-01

Cerebrum

Input ID	*Symbol*	*Name*	**Over/Under representation**
ENSG00000123360	PDE1B	phosphodiesterase 1B, calmodulin-dependent	1.429e-06
ENSG00000176720	BOK	BCL2-related ovarian killer	1.325e-04
ENSG00000137497	NUMA1	nuclear mitotic apparatus protein 1	-4.363e-04
ENSG00000154274	C4orf19	chromosome 4 open reading frame 19	-1.253e-01
ENSG00000184900	SUMO3	SMT3 suppressor of mif two 3 homolog 3 (S. cerevisiae)	1.703e-01
ENSG00000165202	OR1Q1	olfactory receptor, family 1, subfamily Q, member 1	1.747e-01
ENSG00000178015	GPR150	G protein-coupled receptor 150	2.058e-01
ENSG00000152463	OLAH	oleoyl-ACP hydrolase	2.378e-01
ENSG00000145014	TMEM44	transmembrane protein 44	-4.770e-01
ENSG00000149516	MS4A3	membrane-spanning 4-domains, subfamily A, member 3 (hematopoietic cell-specific)	-4.787e-01

Brain

Input ID	*Symbol*	*Name*	**Over/Under representation**
ENSG00000123360	PDE1B	phosphodiesterase 1B, calmodulin-dependent	6.832e-18
ENSG00000154274	C4orf19	chromosome 4 open reading frame 19	-6.731e-04
ENSG00000152463	OLAH	oleoyl-ACP hydrolase	8.185e-03
ENSG00000177291			1.254e-02
ENSG00000137497	NUMA1	nuclear mitotic apparatus protein 1	-8.502e-02
ENSG00000165202	OR1Q1	olfactory receptor, family 1, subfamily Q, member 1	9.962e-02

ENSG00000184900	SUMO3	SMT3 suppressor of mif two 3 homolog 3 (S. cerevisiae)	1.456e-01
ENSG00000145014	TMEM44	transmembrane protein 44	1.479e-01
ENSG00000153029	MR1	major histocompatibility complex, class I-related	1.636e-01
ENSG00000157111	TMEM171	transmembrane protein 171	3.916e-01
ENSG00000176720	BOK	BCL2-related ovarian killer	4.136e-01
ENSG00000178015	GPR150	G protein-coupled receptor 150	5.097e-01
ENSG00000183691	NOG	noggin	5.097e-01
ENSG00000149609	C20orf144	chromosome 20 open reading frame 144	-6.403e-01

Uterus

Input ID	*Symbol*	*Name*	**Over/Under representation**
ENSG00000184900	SUMO3	SMT3 suppressor of mif two 3 homolog 3 (S. cerevisiae)	1.598e-02
ENSG00000123360	PDE1B	phosphodiesterase 1B, calmodulin-dependent	-6.591e-02
ENSG00000176720	BOK	BCL2-related ovarian killer	1.312e-01
ENSG00000165202	OR1Q1	olfactory receptor, family 1, subfamily Q, member 1	1.952e-01
ENSG00000183691	NOG	noggin	2.294e-01
ENSG00000145014	TMEM44	transmembrane protein 44	2.522e-01
ENSG00000152463	OLAH	oleoyl-ACP hydrolase	-2.726e-01
ENSG00000137497	NUMA1	nuclear mitotic apparatus protein 1	3.171e-01
ENSG00000154274	C4orf19	chromosome 4 open reading frame 19	-3.420e-01
ENSG00000153029	MR1	major histocompatibility complex, class I-related	-3.894e-01
ENSG00000157111	TMEM171	transmembrane protein 171	5.804e-01
